# Targeted ferroptosis induction enhances chemotherapy efficacy in chemoresistant neuroblastoma

**DOI:** 10.1038/s41698-025-01090-6

**Published:** 2025-09-16

**Authors:** Adriana Mañas, Alexandra Seger, Aleksandra Adamska, Kyriaki Smyrilli, Joachim T. Siaw, Katarzyna Radke, Erick A. Muciño-Olmos, Oscar C. Bedoya-Reina, Javanshir Esfandyari, Kristina Aaltonen, Daniel Bexell

**Affiliations:** 1 Division of Translational Cancer Research, Department of Laboratory Medicine, Lund University, Lund, Sweden; 2 Translational Research in Pediatric Oncology, Hematopoietic Transplantation and Cell Therapy, IdiPAZ Research Center, University Hospital La Paz, Madrid, Spain; 3 Pediatric Onco-hematology Clinical Unit IdiPAZ-CNIO, National Cancer Research Center (CNIO), Madrid, Spain; 4 Childhood Cancer Research Unit, Department of Women’s and Children’s Health (KBH), Karolinska Institutet, Stockholm, Sweden; 5 School of Medical Sciences, Örebro University, Örebro, Sweden

**Keywords:** Paediatric cancer, Cancer therapeutic resistance, Paediatric cancer, Cancer therapeutic resistance, Chemotherapy

## Abstract

Neuroblastoma (NB) is an aggressive pediatric solid tumor which often develops chemoresistance. Ferroptosis is a potential vulnerability in NB, but its interplay with chemoresistance and standard-of-care chemotherapy is not known. Here, we report that key antioxidant pathways are enriched in refractory NB, and that ferroptosis can be induced in NB through various mechanisms of action (MOA) in vitro and in vivo. We observed that NB standard-of-care chemotherapy can interfere with certain ferroptosis-inducing mechanisms, particularly those targeting GPX4, and that the combination of ferroptosis-inducing drugs with current clinical therapy should be based on MOA. Our work also shows that a combination of chemotherapy and the thioredoxin reductase inhibitor Auranofin counteracted some of the anti-ferroptotic effects of chemotherapy and the combination outperformed chemotherapy alone, resulting in increased survival in a chemoresistant NB patient-derived xenograft model. The combination of Auranofin and chemotherapy decreased the population of immature mesenchymal-like NB cells in vivo and exerted its effect through ferritinophagy, lysosome accumulation and iron overload. Thus, upon careful selection of the MOA, the inclusion of ferroptosis-inducing agents within a clinically relevant treatment protocol is feasible and can outperform standard-of-care chemotherapy in high-risk NB.

## Introduction

Resistance to apoptotic cell death is a common feature in most aggressive and relapsed cancers. This is also observed in high-risk neuroblastoma (HR-NB)^1^, a solid pediatric tumor of the sympathetic nervous system that is driven by large chromosomal aberrations, and is characterized by high intra- and inter-patient heterogeneity and few somatic driver mutations^2,3^. *MYCN* amplification, *CASP8* hypermethylation, and amplification of chromosome 17q, which encodes, amongst others, the anti-apoptotic protein Survivin, are some examples of common NB aberrations that contribute to apoptosis resistance^1,4,5^. Resistance to apoptosis may partly explain the high levels of intrinsic and acquired resistance to chemotherapy observed in HR-NB, because the treatment protocols and chemotherapeutic agents commonly used to treat NB are meant to trigger apoptotic cell death^6^.

*MYCN*-amplified HR-NB tumors, which have high metabolic needs, are dependent on iron and cysteine (key for glutathione production), making them potentially sensitive to ferroptotic cell death^7–9^. Ferroptosis is a form of cell death mediated by labile iron and characterized by the unrestricted accumulation of peroxidized membrane phospholipids, leading to membrane damage^10,11^. Moreover, chemoresistance in NB is associated with a higher presence of cancer stem cells and persister cells, with an immature mesenchymal (MES) phenotype^12–17^. MES cells have been suggested to be vulnerable to ferroptosis, as they often present an upregulated iron metabolism and are dependent of antioxidant pathways^18–20^.

Multiple drugs can induce ferroptosis in cancer cells, directly or indirectly, by various mechanisms^21–23^. However, little is known about which of these agents and mechanisms would be appropriate to target highly heterogenous HR-NB in a clinically relevant setting, such as how they interact with chemotherapy and if they can be implemented as part of the current standard-of-care chemotherapeutic regimes.

In this study, we used *MYCN*-amplified HR-NB patient-derived xenografts (PDXs) and PDX-derived organoid models^12,24,25^ to investigate multiple ferroptosis-inducing agents with different mechanisms of action. The compounds that were effective across multiple models were further tested as single agents and in combination with a clinically relevant protocol mimicking COJEC^12^, the five-chemotherapy cocktail (cisplatin, vincristine, carboplatin, etoposide and cyclophosphamide) that is the first line of action to treat HR-NB patients set by the International Society of Pediatric Oncology–European Neuroblastoma (SIOPEN)^6^. We identified which mechanisms can be exploited to trigger ferroptosis in HR-NB, demonstrated diverse interactions between ferroptosis-inducing compounds and chemotherapy, and showed that ferroptosis induction can be successfully implemented as part of a clinically relevant standard-of-care protocol.

## Results

### High activity of antioxidant pathways correlates with poor prognosis in HR-NB

**Fig. 1:**
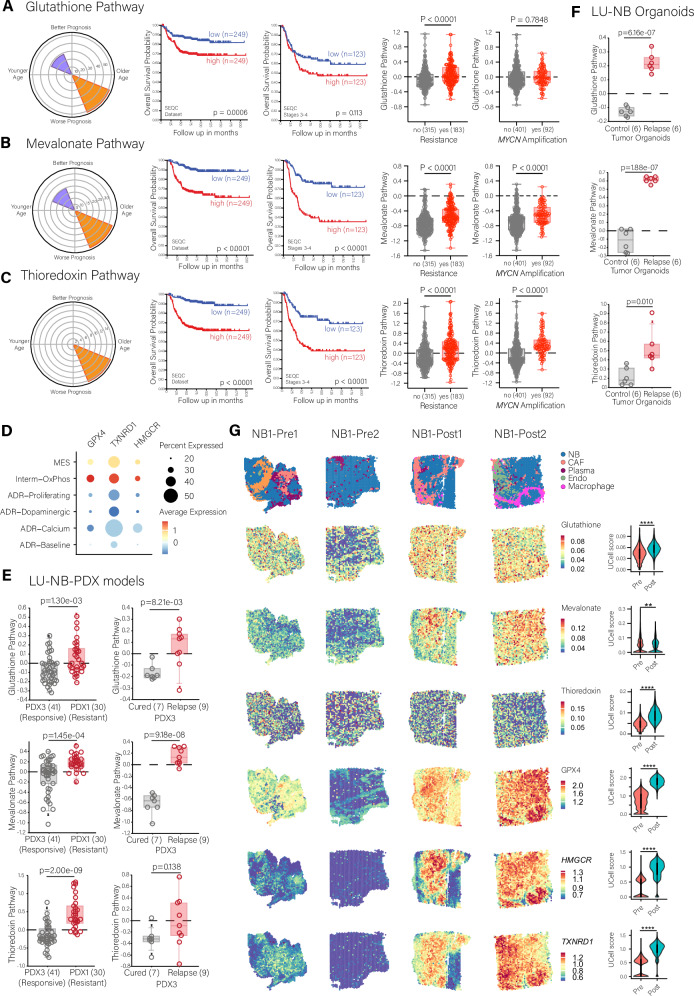
RNA expression of genes involved in ferroptosis-related antioxidant pathways correlates with poor prognosis and treatment resistance in neuroblastoma. **A–C** Analysis of the RNA expression levels of genes involved in the glutathione (**A**), mevalonate (**B**), and thioredoxin (**C**) pathways within the SEQC NB patient dataset (*n* = 498). From left to right: radial graphs correlating expression of the pathway with age and prognosis (age division is younger age <18 months and older age = >18 months), radial increase = -logPvalue); Kaplan Meier overall survival curves for the full patient set and for the high-risk patients (log rank statistical analysis); Box plots comparing RNA expression based on treatment resistance and *MYCN* amplification (t-test with Welch correction statistical analysis). **D** Average gene expression of the enzymes *GPX4*, *TXNRD1* and *HMGCR* in tumor cell populations from a single-nuclei dataset of 22 NB patient tumors. MES mesenchymal, Interm-OxPhos intermediate state enriched in oxidative phosphorylation, ADR adrenergic. **E** Comparison of gene expression levels involved in the glutathione, mevalonate, and thioredoxin pathways between a chemoresistant and a chemosensitive LU-NB PDX model (PDX1 and PDX3, respectively), and between PDX3-derived tumors with a successful response to treatment (cured) and tumors that acquired resistance (relapse). T-test with Welch correction statistical analysis. **F** Comparison of gene expression levels involved in the glutathione, mevalonate, and thioredoxin pathways between PDX3-derived LU-NB organoids established from untreated (Control) and relapsed tumors. T-test with Welch correction statistical analysis. **G** Spatial transcriptomic analysis of an NB patient tumor (NB1) pre- and post-chemotherapy. Samples were analyzed for the expression of genes involved in the glutathione pathway, mevalonate pathway, thioredoxin pathway, and *GPX4*, *HMGCR* and *TXNRD1*. NB neuroblastoma tumor cells, CAF cancer-associated fibroblasts, Plasma plasma cells, Endo endothelial cells. Statistical analysis performed with unpaired two-sided Wilcoxon test. **p-value < 0.01, ****p-value < 0.0001.

To investigate HR-NB vulnerability to ferroptosis, we explored the expression of genes involved in three antioxidant pathways (Fig. 1, Figure S1 and Supplementary data S1): the glutathione pathway, which encompasses the intake of cystine, production of glutathione (GSH) and GSH usage by glutathione peroxidase 4 (GPX4) to reduce lipid peroxides^22^; the mevalonate pathway, which leads to the production of the antioxidant coenzyme Q10 that is essential for the function of the ferroptosis suppressor protein FSP1^26,27^; and the thioredoxin pathway, which involves the enzyme thioredoxin reductase (TrxR) in the process of reducing protein and lipid peroxides^28^. We evaluated the association of high or low expression of genes in these pathways, as an indicator of the activity of the pathways, with patient survival and therapeutic resistance in NB.

We analyzed these three pathways within NB using data from multiple sources: (i) from three large patient cohorts of bulk RNA data publicly available (SEQC [*n* = 498]^29^, Versteeg [*n* = 88]^30^, Kocak [*n* = 476]^31^); (ii) from two single-nuclei transcriptome human datasets^17,32^; (iii) from spatial transcriptomic data from two NB patients pre- and post-chemotherapy^33^; (iv) from multiple conventional cell lines available through the DepMap portal; and (v) from LU-NB (Lund University – Neuroblastoma) PDX models and LU-NB PDX-derived organoid models from our previous work^12^ (Table S1). High expression of these pathways in NB patients significantly correlated with older age (>18 months, indicator of high-risk) and an overall poor prognosis (radial graphs, Fig. 1A–C), and patients with a higher expression (median cut) presented with significantly lower overall survival (Kaplan-Meier curves, Fig. 1A–C and Figure S1A–C), even within the high-risk groups (stages 3–4). Higher activity of these antioxidant pathways also correlated with resistance, either intrinsic or acquired, and with *MYCN* amplification (box plots, Fig. 1A–C and Figure S1A–C). Collectively, high expression of these pathways correlated with high-risk factors and an overall worse survival.

Upon analysis of a single-nuclei dataset of human NB patient tumors^17^, we observed differences in the enrichment distribution of the three antioxidant pathways across tumor cells and patients (*n* = 11) (Figure S1D). The glutathione pathway was active across the largest distribution of patients and across the most types of tumor cells. Activity of the thioredoxin pathway showed an intermediate distribution. The mevalonate pathway activity was high in the smallest subset of tumor cells and patients. We further analyzed the expression of the rate-limiting key enzyme in each pathway (GPX4 for the glutathione pathway, HMG-CoA reductase [encoded by *HMGCR*] for the mevalonate pathway, and TrxR [encoded by *TXNRD1*] for the thioredoxin pathway) in another single-nuclei dataset comprised of 22 NB patient tumors^32^. The analysis showed that cells with MES and intermediate phenotypes had the highest average gene expression of the enzymes of interest (Fig. 1D), suggesting that these cell populations might be vulnerable to ferroptosis induction.

In HR-NB PDX models, the intrinsically chemoresistant LU-NB PDX1 presented an overall higher expression of the antioxidant pathways than the initially responsive LU-NB PDX3 (Fig. 1E). PDX3 tumors treated with COJEC that relapsed or regrew with acquired resistance^12^ had significantly higher levels of the antioxidant pathways compared to PDX3 tumors that were cured (Fig. 1E). Consistently, organoids derived from LU-NB PDX3-relapsed tumors (PDX3-R) (Table S1) had a significantly higher pathway activity than organoids derived from untreated PDX3 tumors (Fig. 1F). These results in patient-derived models indicated that COJEC treatment may modulate the expression of antioxidant pathways.

To validate this in NB patient tumors, we used spatial transcriptomic analysis on paired tumor specimens obtained from two high-risk NB patients before and after treatment (Fig. 1G and Figure S1E; two samples per condition)^33^. Patient NB2 had *MYCN* amplification and *ALK* mutation, whereas NB1 had neither. Both patients were treated with COJEC. Spatial analysis showed that post-treatment samples presented a significantly higher expression of these pathways, and the expression correlated with areas rich in NB cells. In addition, the expression of the genes coding for the three key enzymes GPX4, HMG-CoA and TrxR was significantly upregulated in both patients’ post-treatment samples (Fig. 1F and Figure S1E), further supporting the link between chemotherapy and enhanced antioxidant pathway activity.

To confirm that the pathway activity inferred from gene expression data reflected a dependency of NB on the antioxidant pathways, we assessed the effect of RNA interference (RNAi) knockdown or CRISPR-mediated knockout of the three rate-limiting enzymes. We evaluated data from multiple conventional cell lines across 24 different cancer types^34,35^, including NB. DEMETER2 (for RNAi) and CERES (for CRISPR) scores express how lethal is a knockdown or knockout, with highly negative values indicating stronger dependency of that gene. Both RNAi- and CRISPR-based data indicated that NB is highly dependent on these pathways (Figure S1F).

Thus, our analyses showed that NB is dependent on the glutathione, mevalonate, and thioredoxin pathways, and that higher activity of these pathways correlates with poor survival in NB patients, and chemotherapy resistance. Our findings suggest that targeting these antioxidant pathways could be a promising way of treating HR-NB.

### Ferroptosis can be induced in HR-NB through different mechanisms of action

**Fig. 2:**
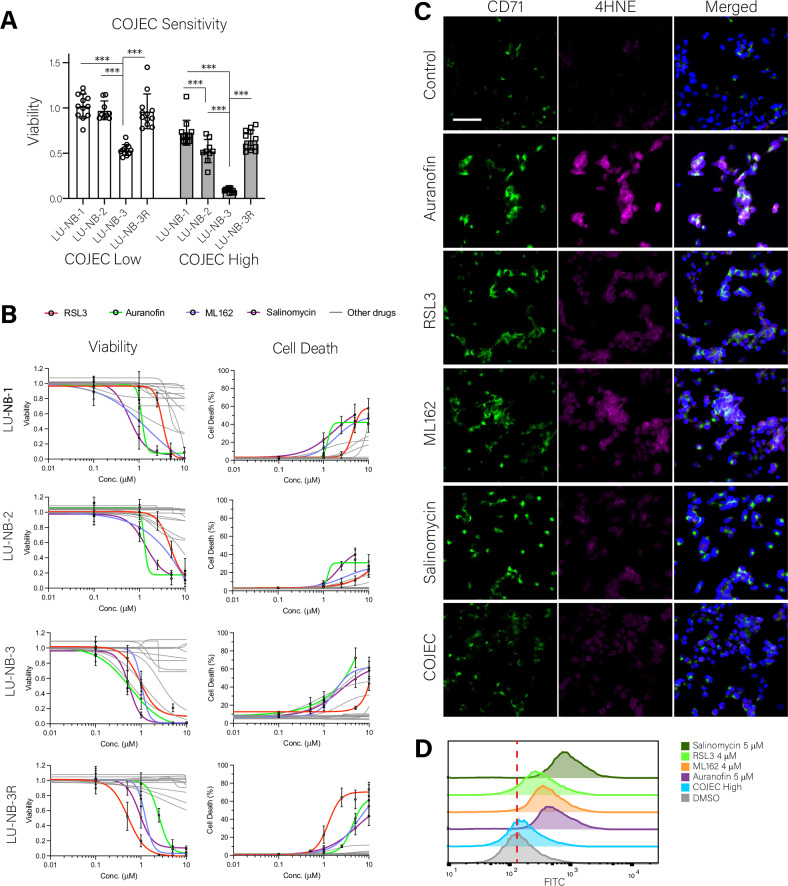
Screening of ferroptosis-inducing compounds across patient-derived xenograft organoid NB models. **A** Analysis of sensitivity to COJEC chemotherapy displayed by the four NB PDX-derived organoid models (LU-NB-1, LU-NB-2, LU-NB-3, and LU-NB-3R). Two different treatments were evaluated (COJEC Low and COJEC High). Statistical analysis with One-way ANOVA and T-test with Tukey multiple comparison correction (***P-value < 0.001). **B** NB cell viability and cell death data for 16 ferroptosis-inducing compounds across the four NB organoid models treated for 48 h. Data for the four most successful drugs across all models are marked in color (RSL3 red, Auranofin green, ML162 blue, Salinomycin purple); the rest are presented in grey. **C** Immunocytofluorescence analysis of ferroptosis markers CD71 and 4HNE for the four selected drugs from (**B**), untreated control, and COJEC chemotherapy in laminin-attached PDX1-derived organoids treated for 24 h. Control 1% DMSO, Auranofin 1 µM, RLS3 1.5 µM, ML162 0.1 µM, Salinomycin 0.25 µM, COJEC High Dose. CD71 = green, 4HNE = magenta, nuclear DAPI staining = blue. Scale bar = 50 μm. **D** Flow cytometry-based analysis of lipid peroxidation for LU-NB-1 organoids treated with the four selected drugs from (**B**), untreated control, and COJEC for 24 h. Control 1% DMSO, Auranofin 5 µM, RLS3 4 µM, ML162 4 µM, Salinomycin 5 µM, COJEC High Dose.

Regulation of ferroptosis is complex, with multiple biochemical processes influencing its occurrence. Thus, multiple mechanisms for triggering ferroptosis are possible. However, not all mechanisms may be equally successful, given the broad inter- and intratumor heterogeneity of HR-NB^2,3^. To identify which mechanisms are promising, we selected 16 compounds that can induce ferroptosis through different mechanisms of action (MOA)^36–50^ (Table 1). We used four LU-NB PDX-derived organoid models that represent primary or relapsed tumors from three different NB patients with *MYCN*-amp (Table S1) and have varied sensitivity to COJEC (Fig. 2A). COJEC is the SIOPEN clinically used combination of cisplatin, vincristine, etoposide, cyclophosphamide, and carboplatin^6^. We observed that most ferroptosis-inducing drugs were effective in at least one model (Fig. 2B and Figure S2), but only four drugs had a consistently strong effect across all models, as determined by the area under the curve (AUC) values for both cell viability and cell death (Table S2). These drugs were: (i) Auranofin, an FDA approved gold-based small molecule that inhibits TrxR^37,51^; (ii) ML162 and (iii) RSL3, which are GPX4 inhibitors^46,48^; and (iv) salinomycin, an antibacterial that alters the autophagic flux thereby promoting iron and ROS accumulation in lysosomes leading to lysosomal membrane disruption^44^. Induction of ferroptosis by each of these four drugs was confirmed through staining for CD71 (Transferrin receptor, an iron transporter) and 4HNE (4-Hydroxynonenal, a product of lipid peroxidation) (Fig. 2C, Figure S3A), which are markers of ferroptosis^52^, and also by analysis of lipid peroxidation with flow cytometry (Fig. 2D). Due to their efficacy across HR-NB models, these four drugs were selected for further experiments.

**Table 1:** Ferroptosis-inducing agents and mechanisms of action

Compound Name	Mechanism of Action	Reference
Altretamine	GPX4 inhibitor	^38^
Artesunate	Iron-dependent ROS production via dihydroartemisin formation, downregulation of GPX4 and inhibition of glutathione S-transferase (GST)	^39^
Auranofin	Thioredoxin Reductase (TRXR) inhibitor	^37^
Buthionine Sulfoximine (BSO)	Inhibition of GSH synthesis	^40^
Cisplatin	Depletion of GSH, model dependent	^41^
Docosahexaenoic acid (DHA)	n-3 PUFA supplementation, enhanced lipid peroxidation	^36^
Erastin	System Xc- inhibitor	^42^
FIN56	Degradation of GPX4 and depletion of coQ10	^43^
Fluvastatin	HMG-CoA reductase inhibitor	^45^
Ironomycin	Upregulation of Tf receptor and iron-mediated peroxidation of lysosomal membranes	^44^
Lovastatin	HMG-CoA reductase inhibitor	^45^
ML162	GPX4 inhibitor	^46^
ML210	GPX4 inhibitor	^47^
(1S,3 R)-Ras Selective Lethal 3 (RSL3)	GPX4 inhibitor	^48^
Salinomycin	Upregulation of Tf receptor and iron-mediated peroxidation of lysosomal membranes	^44^
Sulfasalazine (SAS)	System Xc- inhibitor	^49^
Sorafenib	System Xc- inhibitor	^50^

### Auranofin and RSL3 are effective in chemoresistant HR-NB PDX in vivo

Toxicity tests in vivo were performed with auranofin, ML162, RSL3, and salinomycin in immunocompromised NSG (NOD scid gamma) mice. Salinomycin was not well tolerated, therefore we substituted it by the more potent alkyne-containing derivative ironomycin^44^, which showed excellent efficacy in vitro for LU-NB organoids (Figure S3B). All four drugs were well tolerated (Figure S3C). Despite the lack of toxicity, ML162 was discarded due to poor drug absorption in vivo (Figure S3D). We proceeded to assess in vivo efficacy with auranofin, ironomycin, and RSL3.

**Fig. 3:**
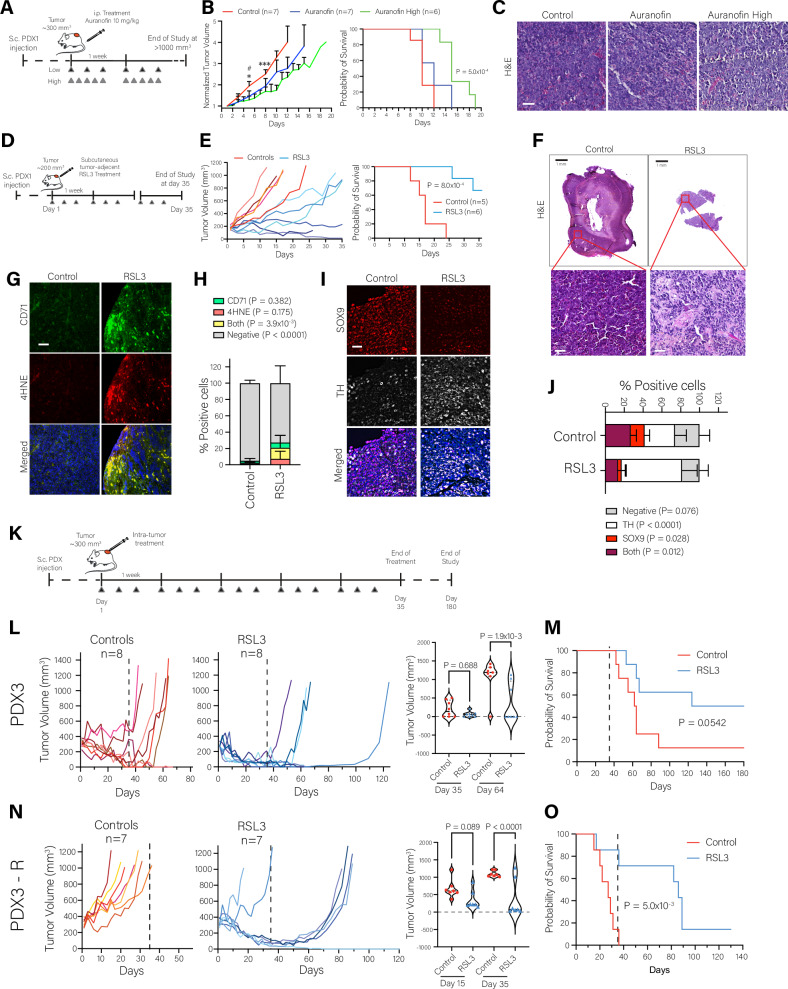
In vivo treatment of a chemoresistant NB PDX with ferroptosis-inducing agents. **A** Schematic summary of the experimental design for Auranofin in vivo treatment. **B** Normalized tumor growth curves and Kaplan Meier survival curves for experiment described in (**A**). Treatment groups: Control (*n* = 7, red), Auranofin (*n* = 7, blue), Auranofin High frequency (*n* = 6, green). Log rank statistical analysis for survival, and mixed-effects analysis multiple comparison for tumor growth (Control-Auranofin, # *P*-value < 0.05; Control-Auranofin High, *P-value < 0.05 and ***P-value < 0.001). **C** Hematoxylin and Eosin (H&E) staining of representative tumors from (**B**). Scale bar 50 µm. **D** Schematic summary of the experimental design for subcutaneous RLS3 in vivo treatment. **E** Tumor volume growth curves and survival Kaplan Meier curves for the control (*n* = 5, red) and RSL3 treated (*n* = 6, blue) groups. Statistical analysis T-test with Welch correction. Log rank statistical analysis. **F** H&E staining of representative tumors from (**E**). Scale bars 1 mm for top panels and 50 µm for bottom panels. **G** Immunofluorescence analysis of ferroptosis markers CD71 and 4HNE in representative tumors from (**E**). CD71 = green, 4HNE = red, nuclear DAPI staining = blue. **H** Quantitative analysis of the CD71 and 4HNE staining in (**G**). A total of 26247 cells were counted across five control tumors and 19307 cells across four treated tumors. Two-way ANOVA statistical analysis followed by multiple comparison with two-stage step-up correction. **I** Immunofluorescence analysis of SOX9 and TH in representative tumors from (**E**). TH = white, SOX9 = red, nuclear DAPI staining = blue. **J** Quantitative analysis of the SOX9 and TH staining in (**I**). A total of 9077 cells were counted across three control tumors and 13194 cells across four treated tumors. Two-way ANOVA statistical analysis followed by multiple comparison with two-stage step-up correction. **K** Schematic experimental design for intratumor RLS3 in vivo treatment. **L** Tumor growth curves and tumor volume comparison at relevant time points for the PDX3 – primary model (PDX3). Controls (*n* = 8, red), RSL3 treated (*n* = 8, blue). One-way ANOVA statistical analysis followed by multiple comparison with Sidak correction. **M** Kaplan Meier survival curve for the treatment groups in (**L**). Log rank statistical analysis. **N** Tumor growth curves and tumor volume comparison at relevant time points for the PDX3 – relapse model (PDX3-R). Controls (*n* = 7, red), RSL3 treated (*n* = 7, blue). One-way ANOVA statistical analysis followed by multiple comparison with Sidak correction. **O** Kaplan Meier survival curve for the treatment groups in (**N**). Log rank statistical analysis.

Mice bearing chemoresistant LU-NB PDX1 tumors were treated with ironomycin (1 mg/kg) for 5 days/week (Figure S3E), but showed no effect on tumor growth or animal survival (Figure S3F). Mice bearing PDX1 tumors were treated with auranofin (10 mg/kg) either 3 days/week or 5 days/week (Fig. 3A). The high frequency administration significantly slowed tumor growth and extended mouse survival (*p* = 5.0 × 10^−^^4^) (Fig. 3B). Furthermore, the tumor tissue showed moderate morphological variations (Fig. 3C).

Two routes of local administration were tested for RSL3: subcutaneous injection adjacent to the tumor and intratumor injection. Mice bearing PDX1 tumors were treated subcutaneously with RSL3 (volume 200 μl, 1.25 mg/injection) 3 days/week for 35 days (Fig. 3D). Subcutaneous administration slowed tumor growth in all RSL3-treated tumors and reduced tumor size in 3/6 tumors (Fig. 3E, left). Survival was also significantly extended, with 4/6 mice surviving the complete treatment period (Fig. 3E, right, *p* = 8.0 × 10^−^^4^). One mouse was euthanized early due to metastatic spread despite significant reduction of the primary tumor. Clear morphological differentiation was seen in most RSL3-treated tumors (Fig. 3F), and a significant proportion of tumor cells were positive for CD71 and 4HNE (Fig. 3G, H, *p* = 3.9 × 10^−3^).

To assess if cells with an undifferentiated or MES-like phenotype were affected, we performed immunostaining for the embryonic marker SOX9 (SRY-box transcription factor 9) and the marker of adrenergic (ADR) neuron differentiation TH (tyrosine hydrolase)^12^. We observed a reduction in the proportion of cells positive for SOX9 and an overall increase in the proportion of cells positive for TH (Fig. 3I, J), indicating that RSL3 could preferentially target NB cells with an embryonic MES-like phenotype or induce a shift towards a more differentiated ADR phenotype.

Given the variable responses observed following subcutaneous administration and the known bioavailability limitations of GPX4 inhibitors, we also tested intratumoral delivery of RSL3 (volume 100 μl, 0.625 mg/injection) (Figure S3G). RSL3 intratumor treatment reduced tumor size below 100 mm^3^ in 6/8 tumors after 4 to 9 injections (Figure S3H). Histological analysis of RSL3-treated tumors revealed pronounced extracellular matrix remodeling and fibrotic tissue reorganization, accompanied by a substantial depletion of viable tumor cells (Figure S3I). These tumors also exhibited a higher proportion of cells positive for 4HNE and CD71 (Figure S3J). RNA sequencing confirmed the robust elimination of tumor cells, with highly damaged RNA extracted from the RSL3 treatment group. Less than 5% of readable transcripts could be mapped exclusively to the human genome in 5/8 RSL3-treated tumors (Figure S3K).

Overall, our results showed that auranofin and RSL3 reduce growth and induce tumor regression, respectively, of established chemoresistant HR-NB tumors as single agents.

### RSL3 can induce long-term remission of HR-NB PDX in vivo

Our data, along with other studies^7,8^, suggested that GPX4 inhibition might effectively target chemoresistant and persister NB cells. Therefore, we tested if GPX4 inhibition with RSL3 could be effective as a first-line therapy (Fig. 3K–O) by treating two paired HR-NB PDX models derived from the same patient (Table S1): LU-NB PDX3 is a primary treatment-responsive model with an overall ADR phenotype, and LU-NB PDX3-R is a chemoresistant relapse model with a primarily MES phenotype^12^. Both models showed a strong response to RSL3 with reduction of tumor size compared to controls and increased survival (Fig. 3L–O; *p* = 5.4 × 10^−2^ and *p* = 5.0 × 10^−3^, respectively). For PDX3, 7/8 tumors were undetectable at the end of the treatment period (day 35) and 4/8 remained undetectable at the end of the experiment (day 180) (Fig. 3L, M). Because some PDX3 controls were affected by intratumor vehicle injection, some of the reduction in tumor size might be attributed to the mechanical damage caused by the intratumor administration. However, controls in PDX3-R were not affected by intratumor vehicle injection (Fig. 3N), so the reduction in tumor size in 5/7 treated tumors can be confidently attributed to RSL3. For PDX3-R no controls survived beyond the treatment period, whereas the median survival for the RSL3-treated group was 86 days and 1/7 RSL3-treated tumors remained undetectable at the end of the experimental time (day 130) (Fig. 3N, O). Thus, RSL3 can induce complete and stable remission in both primary and relapsed HR-NB PDX models.

### Combining RSL3 with chemotherapy is limited by the mechanisms of action

Given the potential of ferroptosis-inducing agents as first-line-of-action treatments, we explored the combination of ferroptosis-inducing drugs with the clinically relevant COJEC induction chemotherapy protocol.

**Fig. 4:**
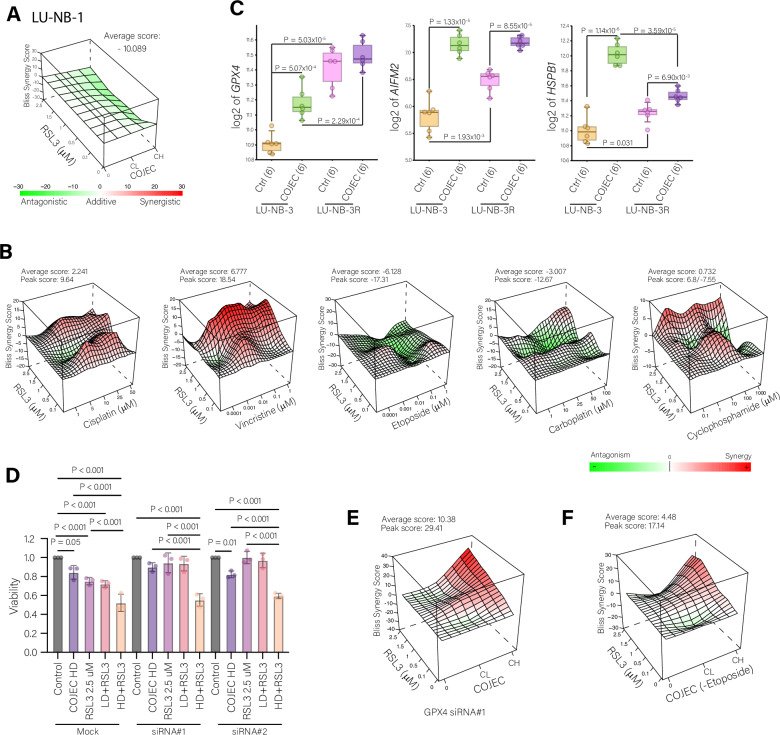
Combination treatment of RSL3 and COJEC chemotherapy. **A** 3D representation of synergy analysis for a dose matrix of RSL3 and COJEC combinations in LU-NB-1 organoids. Bliss analysis performed with SynergyFinder. Negative scores (green) indicate antagonism, positive scores (red) indicate synergy, scores between −5 and 5 are considered additive. Average overall score -10.089. CL = COJEC Low dose; CH = COJEC High dose. **B** 3D representations of synergy analysis for dose matrices of RSL3 combined with the individual COJEC drugs (Cisplatin, Vincristine, Etoposide, Carboplatin, Cyclophosphamide). Bliss analysis performed with SynergyFinder. Negative scores (green) indicate antagonism, positive scores (red) indicate synergy. **C** RNA expression of *GPX4*, *AIFM2* (FSP1), and *HSPB1* in LU-NB PDX3-derived organoids (primary and relapsed), treated or not with high COJEC. One-way ANOVA statistical analysis followed by multiple comparison with Welch’s correction. **D** Viability of LU-NB-1 organoids transfected with GPX4 siRNAs or mock, and treated with DMSO (control), RSL3 2.5 μM, COJEC or the combination. LD low dose, HD high dose. **E** 3D representation of synergy analysis for a dose matrix combination of RSL3 and COJEC in LU-NB-1 organoids that had been transfected with GPX4 siRNA#1. Bliss analysis performed with SynergyFinder. Negative scores (green) indicate antagonism, positive scores (red) indicate synergy. **F** 3D representation of synergy analysis for a dose matrix combination of RSL3 and COJEC without etoposide in LU-NB-1. Bliss analysis performed with SynergyFinder. Negative scores (green) indicate antagonism, positive scores (red) indicate synergy.

We evaluated the effect of low concentrations of RSL3 in combination with either a high or low dose of COJEC on cell viability using the chemoresistant organoid models LU-NB-1 and LU-NB-3R (Figure S4A-B and Table S1). Synergy analysis using SynergyFinder^53^ revealed that RSL3 in combination with COJEC resulted in an antagonistic effect, meaning the combination led to less reduction in viability than what could be expected if the effect of the drugs was just additive, with an average Bliss synergy score of -10.089 for LU-NB-1 and -8.543 for LU-NB-3R (Fig. 4A and Figure S4B).

We hypothesized that this antagonism could be mediated by the multidrug resistance protein 1 (MRP1, encoded by *ABCC1*), a membrane transporter protein of the ATP-binding cassette (ABC) transporter superfamily that is involved in the cellular efflux of multiple compounds, including chemotherapeutic agents^54^. MRP1 has been implicated in the development of multidrug resistance in NB^55^, and can transport vincristine and etoposide out of cells through co-transport with GSH (Figure S4C)^56^. We determined that COJEC treatment increased expression of *ABCC1* in both LU-NB PDX3 models in vivo and in vitro (Figure S4D).

RSL3 can lead to the accumulation of intracellular GSH (Figure S4E), which might enhance drug efflux. Additionally, the accumulation of reduced GSH can inhibit the effect of cisplatin and cyclophosphamide through the formation of less toxic glutathione S-conjugates^41,57^. Previous work showed that MRP1 efflux of GSH promotes ferroptosis^22^. But in this case, in the context of a combination of RSL3 with COJEC, we hypothesized that it might reduce their effectiveness. Therefore, we assessed the intracellular amount of GSH upon the different treatments, individually or in combination (Figure S4E), as well as with buthionine sulfoximine (BSO), a small molecule that depletes intracellular GSH^40^. High-dose COJEC reduced intracellular GSH compared to the DMSO control, whereas RSL3 led to an accumulation of intracellular GSH (Figure S4E). The combination of COJEC and RSL3 resulted in a reduction in GSH similar to that induced by COJEC alone, consistent with any extra GSH that accumulated due to RSL3 inhibition of GPX4 either being pumped out of the cell or binding to components of COJEC. The combination of BSO with RSL3 and COJEC further reduced GSH. However, BSO did not enhance the effect of COJEC (Figure S4F). The combination of RSL3 + BSO or RSL3 + high-dose COJEC + BSO produced similar reductions in viability (Figure S4G, H). Thus, GSH depletion by BSO enhanced the effect of RSL3 but not the effect of COJEC or the combination, indicating that the antagonism involves other processes besides or in addition to those including GSH.

Next, we tested each of the COJEC components individually with RSL3 (Fig. 4B and Figure S4I). The interaction between cyclophosphamide and RSL3 was complex with some concentrations showing a weak antagonism, but with an overall additive profile (Fig. 4B). Cisplatin and vincristine had additive and synergistic profiles respectively when combined with RSL3, whereas carboplatin and especially etoposide had antagonistic interactions with RSL3 (Fig. 4B). Etoposide has been shown to upregulate GPX4^58^ and thus could interfere with the effect of RSL3. By analyzing the effect of COJEC on *GPX4* RNA abundance in LU-NB-3 and LU-NB-3R organoids^12^, we confirmed that COJEC treatment significantly increases *GPX4* expression in NB (Fig. 4C). These findings are consistent with our spatial transcriptomic analysis, which revealed a significant upregulation of *GPX4* mRNA expression in patients’ post-treatment samples (Fig. 1F and Figure S1E). Additionally, we also observed a significant increase in expression of another two genes encoding well-known ferroptosis inhibitors, FSP1 (encoded by *AIFM2*)^27,59^ and heat shock protein beta 1 (encoded by *HSPB1*)^60,61^ (Fig. 4C). To assess the influence of GPX4, we performed partial knock-down (KD) using two different siRNAs (Fig. S4J and Supplementary Data S2). As expected, GPX4 KD completely abrogated the effect of RSL3 (Figure S4K and Fig. 4D), but had no effect on the efficacy of COJEC (Fig. 4D). Upon combination treatment of the siRNA transfected organoids, the effect of RSL3 was restored when combined with high-dose COJEC (Fig. 4D), and the antagonism was counteracted (Fig. 4E). These results are consistent with GPX4 partial restoration by the treatment with COJEC, rescuing the effect of RLS3. To further confirm that the antagonism is mainly mediated by etoposide, we tested RSL3 in combination with COJEC without etoposide, which resulted in an additive effect (Fig. 4F). Overall, these results indicated that COJEC chemotherapy can induce resistance to ferroptosis through chemotherapy-mediated GPX4 upregulation.

### Auranofin shows an additive effect with COJEC chemotherapy

**Fig. 5:**
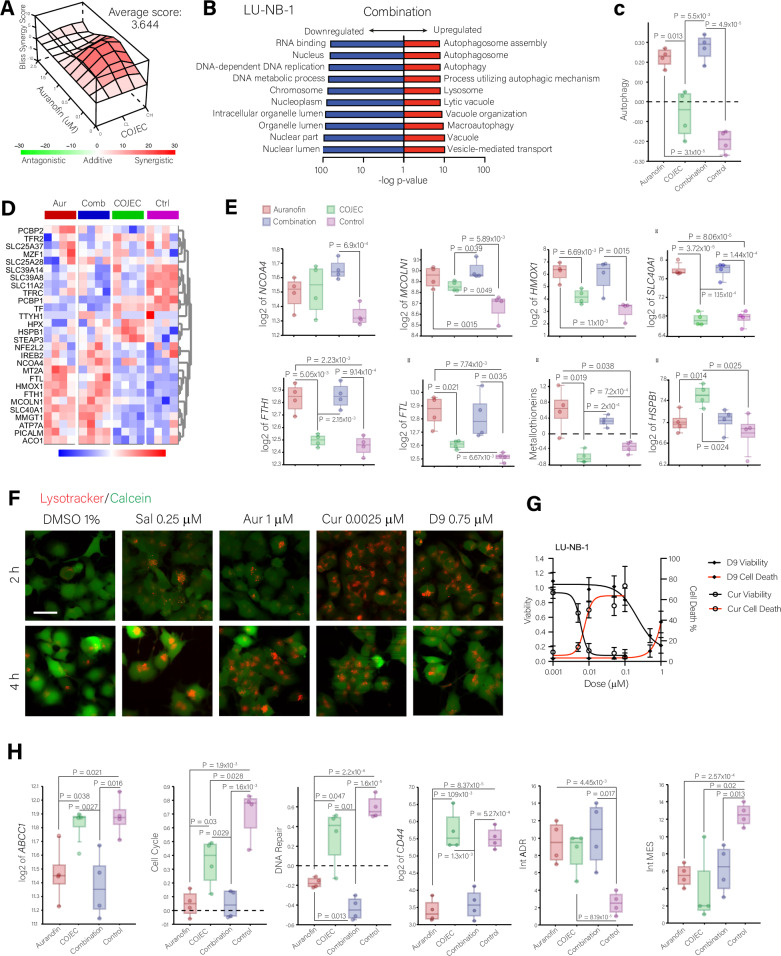
Combination treatment of Auranofin and COJEC chemotherapy. **A** 3D representation of drug synergy analysis for a dose matrix of Auranofin and COJEC combinations in LU-NB-1. Bliss analysis performed with SynergyFinder. Negative scores (green) indicate antagonism, positive scores (red) indicate synergy, scores between −5 and 5 indicate additivity. Average overall score 3.644. CL = COJEC Low dose; CH = COJEC High dose. **B** Gene Ontology analysis of the supervised differentially expressed genes in LU-NB-1 organoids treated with the combination of Auranofin (1 µM)-COJEC High, and controls. Red = Upregulated pathways in the combination group; Blue = Downregulated pathways in the combination group. **C** RNA expression of an autophagy gene signature across treatment conditions (Auranofin, COJEC, Combination and Control). **D**, **E** Supervised analysis of the RNA expression of iron metabolism genes across different treatment groups (log2_z-score range -3 [blue] to 3 [red] and Euclidean distance clustering in (**D**); log2 expression in (**E**). One-way ANOVA statistical analysis followed by multiple comparison with Welch’s correction. **F** Fluorescence staining with Calcein (green) and Lysotracker (Red) in LU-NB-1 organoids treated for 2 and 4 h with DMSO 1%, Salinomycin (Sal) 0.25 µM, Auranofin (Aur) 1 µM, Curcumin (Cur) 0.0025 µM, and D9 0.75 µM. Scale bar 50 µm. **G** NB viability (black) and cell death (red) data after treatment with TrxR inhibitors D9 and Curcumin (Cur) in LU-NB-1 organoids. **H** Supervised analysis of the RNA expression of relevant genes (log2 expression) and signatures (z-score) across treatment groups. One-way ANOVA statistical analysis followed by multiple comparison with Welch’s correction.

Because the antagonism observed between RSL3 and COJEC appears to be mediated by GPX4 upregulation, we decided to test COJEC in combination with a ferroptosis-inducing agent with a different MOA, the TrxR inhibitor Auranofin. The Auranofin-COJEC combination exhibited an additive effect in both LU-NB-1 and LU-NB-3R organoids (Fig. 5A and Figure S4L, M).

We confirmed that Auranofin inhibits TrxR (Figure S4N) and we performed whole transcriptome analysis (RNA-seq) for each PDX-derived organoid model to further explore the mechanisms of the combination. Principal component analysis (PCA) showed clear clustering by model and by treatment (Figure S5A), with the COJEC group clustering close to the controls and the Auranofin-treated samples clustering with the combination. Upon unsupervised analysis of the top 1000 genes exhibiting the greatest change in expression for each model, we observed clear gene clustering by treatment (Figure S5B). Gene Ontology (GO) analysis for Biological Process revealed that the Auranofin and combination groups were dominated by autophagy and vesicle organization and transport pathways (Figure S5B). Supervised differential gene expression analysis and gene ontology comparison between the combination and control groups revealed an upregulation of pathways associated with autophagy in the combination group for both models (Fig. 5B and Figure S5C). Analysis of the expression of an autophagy-specific gene list showed significantly higher expression in the Auranofin and combination groups for both models (Fig. 5C, Figure S6A and Supplementary data S1), indicating that Auranofin is the main mediator of the increase in autophagy.

### Auranofin modulates ferritinophagy to induce ferroptosis in NB

Ferritinophagy is a specific type of autophagy that can lead to iron overload and ferroptosis^62–66^. Expression analysis of genes involved in iron metabolism and ferritinophagy indicated that the Auranofin and combination groups could be promoting this process (Fig. 5D, E, Figure S6B, C and Supplementary data S1). Specifically, we detected higher expression of the ferritinophagy mediator *NCOA4* (encoding nuclear receptor coactivator 4, which binds to ferritin and delivers it to the autophagy-lysosomal machinery)^63^, as well as *MCOLN1* (encoding mucolipin transient receptor potential cation channel, also known as TRPML1), which transports iron from the lysosomes to the cytoplasm^67^ and is required for lysosomal biogenesis^68^ (Fig. 5D, E and Figure S6B, C). Moreover, we observed a pattern of gene expression consistent with the response to an iron overload, such as the upregulation of *HMOX1* (encoding heme oxygenase 1)^69^, *SLC40A1* (encoding ferroportin), *FTH1* and *FTL* (encoding ferritin heavy and light chains, respectively)^70^, and metallothioneins^71^ (Fig. 5D, E, Figure S6B, C and Supplementary data S1). Given that the RNA-seq data strongly pointed to an iron overload mediated by ferritinophagy (Fig. 5B−E), we analyzed lysosome formation under treatment with Auranofin. Consistently, we observed lysosome accumulation within 2 to 4 h of treatment with Auranofin (Fig. 5F).

To test if the ferritinophagy-associated effect is an off-target mechanism of Auranofin or a consequence of general TrxR inhibition, we treated LU-NB-1 organoids with other TrxR inhibitors with different chemical structures. Like Auranofin, D9 (C_25_H_20_AuOPS) is a gold-based compound; whereas Curcumin is a polyphenol^72^. Both drugs were cytotoxic at sub-micromolar concentrations (Fig. 5G and Figure S6D) and induced strong lysosome accumulation at 2 to 4 h of treatment (Fig. 5G) and a decrease in calcein signal compared to the DMSO control, indicative of a higher presence of free intracellular iron.

Additionally, COJEC alone triggered an increase in expression of the ferroptosis inhibitor *HSPB1* in LU-NB-1 (Fig. 5E), and in the LU-NB-3 and -3R models it either increased or maintained *HSPB1* expression (Fig. 4E and Figure S6C) compared to controls. Auranofin alone or in combination with COJEC, significantly reduced *HSPB1* expression (Fig. 5E and Figure S6C), counteracting the effect of COJEC. COJEC alone also increased *ABCC1* expression (encoding MRP1), and Auranofin countered this effect by blocking or limiting the COJEC-mediated increase (Fig. 5H and Figure S6E). We also observed reduced expression of genes involved in the cell cycle and DNA repair in the Auranofin and combination groups (Fig. 5H and Figure S6E).

Moreover, Auranofin reduced the expression of the stem cell marker *CD44*^73^, and impaired the COJEC-induced increase in *CD44* expression (Fig. 5H and Figure S6E). CD44 mediates iron endocytosis enabling iron-mediated epigenetic plasticity^74^, and a variant of CD44 interacts with and stabilizes the glutamate-cystine antiporter Xc, thereby maintaining glutathione synthesis^75^. Thus, this data suggests that downregulation of *CD44* by Auranofin could target NB cells with a MES-like phenotype. Consequently, we analyzed three sets of publicly available ADR or MES gene signatures (Supplementary data S1): the Integrated (Int) ADR signature and Int MES signature^12^, which integrate six established signatures from different sources (cells lines, PDXs, and patient tumors); the ADR, MES, or sympathoadrenal (SYMP) signatures described by Patel et al. that were derived from 55 NB tumors^76^; and the Van Groningen signatures, derived from conventional serum-grown cell lines in vitro^13^. The Van Groningen signatures yielded inconclusive results (Figure S6F). On the other hand, the results for the signatures including patient NB material, the Int and Patel signatures, showed a significant downregulation of MES genes in the Auranofin and combination groups when compared to either the control (Fig. 5H and Figure S6E-F) or the COJEC group (Figure S6F). Results showed that the combination of Auranofin and COJEC is capable of impairing the COJEC-induced MES upregulation seen with the Patel et al. signatures (Figure S6F). The Int signatures also showed an upregulation or maintenance of ADR genes expression in the Auranofin and combination groups compared to the control (Fig. 5H and Figure S6E). Altogether, these results suggest that Auranofin impairs the expression of genes associated with the MES phenotype.

Collectively, the data indicates that the combination of Auranofin and COJEC is useful to target HR-NB cells. Auranofin countered several COJEC-mediated changes that could limit chemotherapy effectiveness, providing mechanisms for a successful combination. Furthermore, TrxR inhibition influenced lysosome accumulation, resulting in iron overload and cell death.

### Combination of Auranofin and COJEC increases survival in a chemorefractory PDX model of HR-NB

**Fig. 6:**
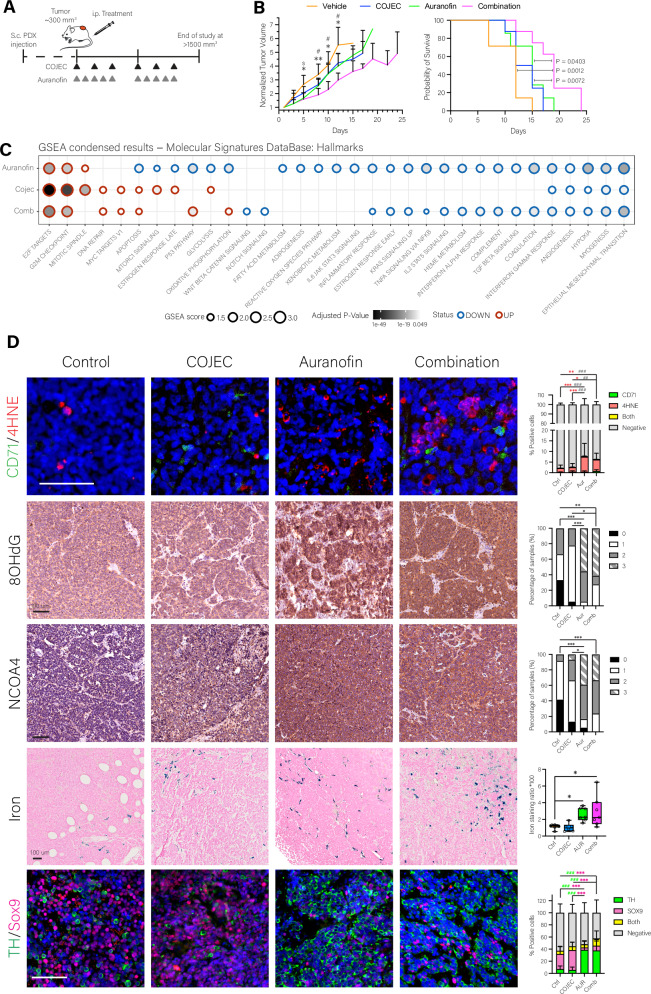
In vivo treatment of NB PDX models with a combination of Auranofin and COJEC. **A** Schematic summary of the experimental design for the Auranofin in vivo treatment. **B** Normalized tumor growth curves and survival Kaplan Meier curves for experiment described in (**A**). Treatment groups: Control (*n* = 7, orange), Auranofin (*n* = 7, green), COJEC (*n* = 8, blue), Combination (*n* = 8, pink). Log rank statistical analysis for survival, and mixed-effects analysis multiple comparison for tumor growth (Control-Auranofin $ *P*-value < 0.05; Control-Combination, *P-value < 0.05, and ** *P* value < 0.01; COJEC-Combination # *P*-value < 0.05). **C** GSEA analysis of RNA expression signatures from tumors from (**B**). Comparison of treatment groups against the control group using the Hallmarks signature database. **D** CD71 (green) and 4HNE (red) fluorescence immunostaining images and quantitation, two-way ANOVA analysis with multiple comparison with Tukey correction (4HNE positive cells comparison: *P-value < 0.05, **P-value < 0.01, ***P-value < 0.001; Negative cells comparison: ^##^*P*-value < 0.01, ^###^*P*-value < 0.001). Oxidative stress marker 8-hydroxy-2’-deoxyguanosine (8OhdG) DAB immunostaining images and quantitation, non-parametric Kruskal-Wallis test with multiple comparison with Dunn’s test correction (*P-value < 0.05, **P-value < 0.01, ***P-value < 0.001). NCOA4 DAB immunostaining images and quantitation, non-parametric Kruskal-Wallis test with multiple comparison with Dunn’s test correction (*P-value < 0.05, **P-value < 0.01, ***P value < 0.001). Prussian Blue iron staining images and quantitation, one-way ANOVA analysis with multiple correction comparison with Dunnett correction (*P-value < 0.05). TH (green) and SOX9 (magenta) fluorescence immunostaining images and quantitation, two-way ANOVA analysis with multiple comparison with Tukey correction (SOX9 positive cells comparison: ***P-value < 0.001; TH positive cells comparison: ^###^*P*-value < 0.001). All scale bars represent 100 µm.

Finally, we tested the combination of COJEC and Auranofin in vivo by treating LU-NB PDX1-bearing mice with the COJEC protocol^12^, the 5-day/week Auranofin regime, and the combination of both (Fig. 6A). Toxicity of the combination therapy was equivalent to that of COJEC alone, as determined by general health assessment and weight loss (Figure S7A), with one mouse from each of those groups excluded from the tumor growth analysis (but not from survival analysis) due to severe weight loss and early humane endpoint. The combination of COJEC and Auranofin resulted in significantly slower tumor growth and increased survival in this chemoresistant HR-NB PDX model (Fig. 6B).

RNA-seq and transcriptomic analysis revealed that Auranofin and the combination with COJEC significantly downregulated pathways associated with tumor malignancy and metastatic capacity, such as MTORC1 signaling, glycolysis, Notch signaling, KRAS signaling, WNT-β catenin signaling, hypoxia, angiogenesis, IL-6/JAK/STAT3 signaling, and epithelial-to-mesenchymal transition (Fig. 6C). Auranofin also downregulated pathways related to lipid, iron, and antioxidant metabolisms (fatty acid metabolism, adipogenesis, heme metabolism, ROS pathways), suggesting that Auranofin could eliminate or differentiate aggressive cancer cells with high lipid, iron, and antioxidant requirements (Fig. 6C). Using GO and Reactome enrichment analysis we also observed that the control group was defined by pathways related to mesenchymal cell proliferation and blood vessel formation, whereas the combination group was enriched in pathways related to metallothioneins and response to metal ions (Figure S7B-C).

Histological analysis showed a small but significant increase in 4HNE-positive cells in the Auranofin and combination groups (Fig. 6D, CD71/4HNE), along with a significant increase in oxidative damage as determined by the staining of the oxidative stress marker 8-hydroxy-2’-deoxyguanosine (Fig. 6D, 8OHdG). The Auranofin and combination groups presented a higher amount of NCOA4 (Fig. 6D, NCOA4) and an accumulation of iron deposits (Fig. 6D, Iron), consistent with ferritinophagy-modulation. Finally, a shift from an embryonic MES-like phenotype (characterized by abundant SOX9) towards an ADR-like phenotype (characterized by abundant TH) seemed to occur in the Auranofin and combination groups (Fig. 6D, TH/Sox9). All these effects appear to be primarily mediated by Auranofin, as no significant histological difference was observed between the Auranofin and combination groups, indicating that the effect of Auranofin is not hindered by the combination with COJEC in vivo.

Thus, we concluded that Auranofin can be successfully combined with a clinically relevant COJEC protocol in vivo, resulting in delayed tumor growth and increased survival in a chemoresistant HR-NB PDX model. This beneficial effect was associated with lipid peroxidation, iron accumulation, and oxidative damage, inducing a combination of ferroptotic cell death and a phenotypic switch towards a more differentiated NB cell state.

## Discussion

We explored ferroptosis-induction in combination with standard-of-care chemotherapy as a treatment option to overcome resistance in HR-NB. We showed that ferroptosis-related antioxidant pathways are dominant in NB tumors with poor prognosis. Using NB organoids, we evaluated a collection of ferroptosis-inducing agents covering different pathways and identified drugs with effect across multiple models. We further explored combinations of ferroptosis-inducing agents with COJEC chemotherapy and identified differential responses, either antagonistic or additive, depending on the drug and MOA. The combination of RSL3 with COJEC resulted in antagonism due to the upregulation of GPX4 and other ferroptosis inhibitors, which was primarily mediated by etoposide. In contrast, the combination of the TrxR inhibitor Auranofin with COJEC had an additive effect that involved ferritinophagy-mediated lysosome accumulation and iron overload, reversing the COJEC-induced MES phenotype.

Our work indicates that ferroptosis induction can be a promising approach to treat chemoresistant HR-NB and, when combined with chemotherapy, it can outperform the current standard-of-care COJEC chemotherapy protocol. However, there are multiple ferroptosis-inducing compounds available with different MOAs, some of which can affect the compatibility with chemotherapy. Furthermore, not all mechanisms are equally effective across patients and models, leading to important considerations for future personalized clinical applications.

The rationale for inducing ferroptosis in HR-NB is multifold: i) NB is often resistant to apoptotic cell death^1^; ii) high levels of serum ferritin correlate with poor prognosis in HR-NB^77,78^; and iii) targeting the glutathione pathway has shown encouraging results^7,8,79^. Here, we explored targeting key antioxidant pathways to induce ferroptosis, as we observed a prognosis-correlated high expression across multiple NB patient cohorts. Our results with multiple preclinical models point to induction of ferroptosis as a promising strategy to target primary NB tumors as well as relapsed and chemoresistant disease, as sensitivity to ferroptosis remains even in models with acquired chemoresistance. Our data also indicates that the inclusion of these compounds in a first-line-of-action protocol might prevent relapses. Critically, our work shows that compounds must be carefully selected based on their MOA and evaluated for inclusion within a chemotherapeutic protocol.

In the context of highly heterogenous tumors like NB, the variety of drugs and MOAs that have been shown to induce ferroptosis enhances the chances of a successful drug-patient personalized match. Although RSL3 and Auranofin were identified as promising agents in our study, we applied a strict threshold to reduce the number of drugs to study in detail. Thus, other non-selected compounds, such as Erastin or Artesunate, could be worth further exploration.

GPX4 inhibition using RSL3 is a successful anti-tumor agent in vitro, but its limited bioavailability makes it challenging to use in vivo. Promising local administration mechanisms have been developed for RSL3^80^, but the current lack of systemic effect renders RSL3 and other GPX4 inhibitors ineffective for targeting metastases or difficult-to-access tumor sites for the moment. Moreover, we showed that antagonism with some common chemotherapeutics makes inclusion of GPX4 inhibitors in conventional treatment protocols challenging, because some chemotherapeutic agents can interfere with ferroptosis. Additionally, the effect of individual chemotherapeutics on ferroptosis may be context dependent. Cisplatin has been proposed to trigger ferroptosis^41^, but at the same time platinum compounds, like cisplatin and carboplatin, can have an iron depleting effect^81^. Although detrimental for cell replication, moderate iron deficiency does not necessarily induce cell death and could render cells quiescent and resistant, which can lead to tumor regrowth upon treatment removal. This is consistent with our results, as cisplatin doesn’t seem to trigger ferroptosis in NB, and with the concept of drug-tolerant persister cells that are induced by cisplatin in NB^82^ and exhibit a transition toward a MES-like cell state^12,13^.

Etoposide is a chemotherapeutic drug that yielded an antagonistic effect with RSL3 in our setting. Etoposide upregulates GPX4 in other tumors^58^, and we observed a similar upregulation of the ferroptosis inhibitors GPX4, FSP1, and HSPB1 in our NB models. Thus, certain chemotherapeutic agents can indirectly inhibit ferroptosis. Nevertheless, Etoposide can still work successfully with other ferroptosis-inducing drugs with different MOAs, such as Erastin^83^ and Auranofin^84,85^, pointing to MOA selection as the key factor to avoid antagonism. Indeed, it is lack of antagonism and not necessarily synergy that should be the final goal of combinations, since synergy is not required for successful combination treatments in patients^86,87^. Future work should address if removal or substitution of certain drugs in the COJEC chemotherapy protocol could lead to better combinations. Nevertheless, given the current challenges with GPX4 inhibitors, we considered it more relevant to continue exploring other ferroptosis-inducers that may be more feasible to combine with chemotherapy.

We also found that Auranofin, a gold-based FDA-approved drug previously used to treat arthritis and known to inhibit TxrR^37,51^, can trigger ferroptosis in chemoresistant HR-NB models and it can be successfully combined with COJEC chemotherapy. We identified lysosomal iron overload, probably induced through ferritinophagy^62,63^, and phenotypic differentiation as the mechanisms by which Auranofin targets NB cells. These mechanistic findings are in line with results from other tumor types. Borneol, an organic terpene derivative, presents anti-tumor activity in combination with cisplatin by triggering ferroptosis through NCOA4-mediated ferritinophagy, also leading to decreased expression of MES markers and enhanced expression of epithelial markers in non-small cell lung cancer (NSCLC) models^88^. Auranofin also enhances *FTH1* and *FTL* expression, increases HMOX1 and reduces GPX4 in NSCLC models^89^, in line with what we observed in NB. Additionally, other TrxR inhibitors, such as Curcumin, have been associated with perturbations in the autophagy-lysosomal degradation pathway^90^ and can reduce GPX4 and FSP1 protein levels^91,92^. Finally, we have seen that the downregulation of *HSPB1*, *CD44*, and *ABCC1* (encoding MRP1) could also contribute to the successful combination of Auranofin with COJEC in NB. Altogether, this indicates that agents that induce ferroptosis through TrxR inhibition might affect multiple pathways or proteins in addition to the thioredoxin pathway, and these could contribute to anti-tumor effects and compatibility with chemotherapy.

Our work shows the importance of the mechanism and molecular context when selecting ferroptosis-inducing compounds to combine with chemotherapy, but the study presents some limitations. Only *MYCN*-amplified models were used for experimental validation, but the patient data indicates that non-*MYCN*-amplified NBs could also be targeted with this approach. Although our results indicate a phenotypic transition consistent with previous reports using similar compounds^44,90^, our study of the MES-ADR transition remains limited. Future studies should expand on this work by incorporating additional markers and signatures, and evaluate a wider range of ferroptosis-inducing drugs to further clarify the impact of ferroptosis in NB phenotypic plasticity in the context of chemotherapy. Moreover, only two compounds were tested in combination with chemotherapy, based on their effectiveness as single agents in vivo. Future studies should also explore additional ferroptosis-inducing drugs in combination with chemotherapy, even if these drugs show limited efficacy as monotherapies. Auranofin is approved for clinical use and is currently being explored for repurposing in cancer (NCT01419691, NCT01747798, NCT02770378, NCT03456700, NCT01737502). However, it is not clear if the doses we used in our study are comparable to clinically achievable doses in pediatric patients.

Most ferroptosis-inducing agents currently in clinical trials for cancer are aimed at the glutathione pathway^93^, specifically at the inhibition of the Xc- system or key enzymes to deplete GSH. The lack of success of BSO, as well as other GSH-depleting drugs that we tested, either alone or when combined with COJEC, indicated that NB cells have compensatory mechanisms to overcome the toxicity of GSH depletion and to mediate treatment resistance. This could explain why clinical trials combining BSO and melphalan to treat refractory high-risk NB have shown very modest results^94,95^. Future work should address the specific role of GSH and its clinical relevance in NB, because targeting other pathways that are not GSH-centric might be more successful.

Our results strongly highlight the importance of carefully identifying the optimal ferroptotic mechanism when selecting drugs for combination therapies. Overall, our work shows the potential of therapeutically targeting ferroptosis in HR-NB and demonstrates both the feasibility and the challenges of including ferroptosis-inducing agents as part of a clinically relevant treatment protocol.

## Methods

### NB organoid culture

LU-NB organoids were previously established from LU-NB PDXs 1, 2, 3 and 3-Relapsed (Table S1)^12,24,25^ and cultured as free-floating 3D organoids under serum-free conditions in low glucose Dulbecco’s modified Eagle’s medium (ThermoFisher, 21885) and GlutaMAX F-12 Nut Mix (ThermoFisher, 31765027) in a 3:1 ratio, supplemented with 1% penicillin/streptomycin (Corning, 30-002-CI), 2% B27 without vitamin A (ThermoFisher, 12587001), fibroblast growth factor (40 ng/ml) (Peprotech, AF-100-15), and epidermal growth factor (20 ng/ml) (Peprotech, AS-100-18B). The identity of the LU-NB cells was confirmed using short tandem repeat (STR) and single nucleotide polymorphism (SNP) analysis.

### Treatment compounds

Ferroptosis-inducing agents: Altretamine (Sigma-Aldrich, 549835), Artesunate (Santa Cruz Biotechnology, sc-201329), Auranofin (Sigma-Aldrich, A6733), Buthionine Sulfoximine (Merck Milipore, 5.08228.001), Curcumin (Sigma-Aldrich, C1386), Docosahexaenoic acid (Santa Cruz Biotechnology, sc-200768), D9 (Sigma-Aldrich, 532910), Erastin (Sigma-Aldrich, E7781), FIN56 (Sigma-Aldrich, SML1740), Fluvastatin (Sigma-Aldrich, 93957-55-2), Ironomycin^44^, Lovastatin (Sigma-Aldrich, PHR1285), ML162 (Sigma-Aldrich, SML2561), ML210 (Sigma-Aldrich, SML0521), (1S,3 R)-Ras Selective Lethal 3 (Sigma-Aldrich, SML2234), Salinomycin (Santa Cruz Biotechnology, sc-253530), Sulfasalazine (Sigma-Aldrich, S0883), Sorafenib (Santa Cruz Biotechnology, sc-220125).

COJEC drugs: cisplatin (Santa Cruz Biotechnology, sc-200896), vincristine (Santa Cruz Biotechnology, sc-201434), etoposide (Santa Cruz Biotechnology, sc-3512), cyclophosphamide (Santa Cruz Biotechnology, sc-361165), and carboplatin (Santa Cruz Biotechnology, sc-202093A). COJEC low dosage: Cisplatin 0.5 μM, Etoposide 0.001 μM, Vincristine 0.001 μM, Cyclophosphamide 5 μM, Carboplatin 0.5 μM. COJEC high dosage: Cisplatin 1 μM, Etoposide 0.01 μM, Vincristine 0.005 μM, Cyclophosphamide 10 μM, Carboplatin 5 μM.

### Cell viability and cell death assays

Cell viability and death were measured using the CytoTox-Glo Cytotoxicity Assay (Promega). Organoids were dissociated using Accutase (Sigma-Aldrich, A6964), and 5000 single cells were seeded per well in white 96-well plates with clear bottom (Corning, 2610). Cells were incubated for 48 h before treatment to allow the formation of small organoids. Treatments were performed in sets of at least 2 technical replicates and at least 3 biological replicates. Luminescence was detected after 48 h of treatment using a Synergy2 plate reader (BioTek). Viability shown as ratio to control and cell death shown as percentage of death per condition.

### Lipid peroxidation assay

Dissociated organoids were seeded with 1 × 10^6^ cells in T-25 flasks. After 48 h, organoids were treated either with: Auranofin (5 μM), Salinomycin (5 μM), RSL3 (4 μM), ML162 (4 μM), COJEC (cisplatin 0.5 μM, vincristine 0.005 μM, etoposide 0.01 μM, cyclophosphamide 10 μM, carboplatin 5 μM), or DMSO (Sigma-Aldrich, D2438). Treated organoids were harvested after 24 h and stained using a Lipid Peroxidation Assay Kit (Abcam, 243377) according to protocol. The stained cells were then frozen at −20 °C until readout using the FACS Melody (BD Biosciences). Results were analyzed with FlowJo.

### Cell immunofluorescence

Dissociated organoids were seeded on glass slides pre-coated with LN521-05 laminin (10 μg/ml; Biolamina). Cells were allowed to attach and grow for at least 72 h, and then they were fixed using 4% paraformaldehyde. Permeabilization and blocking were performed with 0.25% Triton X-100 and 2.5% fetal bovine serum in 1× tris-buffered saline (pH 7.6). The primary antibodies used were against 4HNE (1:50, Abcam, ab46545) and CD71 (1:50, Santa Cruz Biotechnology, sc-32272). The secondary antibodies used were Alexa Fluor 488 (1:200; Invitrogen, ref. A11001) and Alexa Fluor 633 (1:200; Invitrogen, ref. A21071). Nuclear staining was performed with 4′,6-diamidino-2-phenylindole (DAPI; Invitrogen, ref. D3571) for 10 min. Slides were then mounted using a water-based mounting medium. Imaging was performed using an Olympus fluorescent microscope and CellSens software.

### Animal experiments

All in vivo procedures were conducted according to the guidelines from the regional Ethics Committee for Animal Research, Skane, Sweden (permit nos. M11- 15 and 19012–19). Only female mice were used due to ethical considerations for long term housing of males in studies using slow-growing PDXs. NB PDX dissociated organoids (2 × 10^6^ cells) were suspended in a 100 μl mixture (2:1) of stem cell medium and Matrigel (Corning, catalog no. 354234) and injected subcutaneously into the flanks of female NSG mice obtained from in-house breeding. Tumor size was measured using a digital caliper and calculated with the formula V = (πls2)/6 mm^3^ (l, long side; s, short side of each tumor). Mice were randomly allocated to the corresponding treatment groups when tumors reached the appropriate starting size for each experiment. All mice were regularly monitored for weight loss and other signs of toxicity based on general well-being factors such as fur appearance or signs of pain. Mice were euthanized by cervical dislocation based on tumor size, weight loss, overall health deterioration, or end of study time. ML162 and RSL3 were administered subcutaneously or intratumorally, as they are known to have poor solubility and therefore limited systemic effect^48^; auranofin, ironomycin and salinomycin were administered by intraperitoneal injection. Auranofin was dissolved to 2.5 mg/ml in saline with 5% DMSO (Sigma-Aldrich, D2438) and mice were treated with a dose of 10 mg/kg either 3 times/week or 5 times/week. Ironomycin was dissolved to 0.394 mg/ml in saline with 5% DMSO and mice were treated 5 times/week with a dose of 1 mg/kg. Salinomycin monosodium salt was dissolved to 2.5 mg/ml in saline with 5% DMSO and tested for toxicity at 1, 5 and 10 mg/kg. ML162 was dissolved to 5 mg/ml in 2:3 Saline:PEG400, and 200 μl were administered subcutaneously. RSL3 was dissolved to 6.25 mg/ml in 1:1 Saline:PEG400; the subcutaneous dose was 200 μl injection volume, and the intratumor dose was 100 μl injection volume. Control mice were injected intratumorally with 100 μl of vehicle (1:1 saline:PEG-400). COJEC drugs were dissolved in saline and administered by intraperitoneal injections of cisplatin (1 mg/kg) and vincristine (0.25 mg/kg) on Mondays, etoposide (4 mg/kg) and cyclophosphamide (75 mg/kg) on Wednesdays, and carboplatin (25 mg/kg) on Fridays. When Auranofin and COJEC were administered on the same day, they were injected sequentially (COJEC first), allowing at least 2 h in between treatments to reduce animal discomfort.

### Immunohistochemistry and histological analyses

Tumors were fixed in 4% formalin, embedded in paraffin, and cut in 4 μm sections for staining. H&E (Histolab Products) staining was performed for histopathological analysis. DAB (3,3’-Diaminobenzidine) and fluorescence staining were performed manually. Heat-induced antigen retrieval was done using sodium citrate buffer (pH 6.0). The antibodies recognized 4HNE (1:50, Abcam, ab46545), CD71 (1:50, Santa Cruz Biotechnology, sc-32272), 8OHdG (1:1000, Santa Cruz Biotechnology, sc-66038), NCOA4 (1:50, Abcam, ab86707), TH (1:1000, Abcam, ab112), SOX9 (1:500, Abcam, ab76997). Prussian blue iron staining was performed using the Hematognost Fe kit (Sigma-Aldrich, REF1.12084.0001). Processing and quantification of bright field and fluorescence images were performed using ImageJ and QuPath 0.2.3. Semiquantitative analysis of 8OHdG and NCOA4 staining was performed by categorical classification (Negative to High) based on staining intensity perception by three blinded researchers. All slides were scanned using the NanoZoom scanner (Hamamatsu).

### TrxR activity detection

Dissociated LU-NB organoids were seeded in 6-well plates at a concentration of 1 million cell/well and incubated for 48 h. Treatment was applied using 1 µM of Auranofin. Organoids were harvested after 0 h, 4 h, 8 h, and 16 h. After harvesting and washing with 1xDPBS without CA and Mg (Corning, 21-031-CV), RIPA buffer (Thermofisher, #89900) supplemented with a protease inhibitor (cOmplete, Roche, #11697498001) was added and the lysate was incubated for 30 min at 4 °C. The mixture was centrifuged at 14,000 rpm for 15 min at 4 °C. Using the Pierce^TM^ BCA Protein Assay Kit (Thermofisher, #23225) and the associated protocol, protein concentrations were measured using the Synergy2 plate reader (BioTek). TrxR activity was measured using the Thioredoxin Reductase Activity Assay kit (Merk, CS170). A single measurement was taken after 60 min incubation in room temperature using a Synergy2 plate reader (BioTek).

### Live cell imaging

Organoids were dissociated into single cells as described above and grown on laminin (10 ug/ml; LN521-05, BioLamina) coated 48 well plates at a concentration of 0.1 million cells per well for 48 h. Next, cells were stained 1:2400 with LysoTrackerTM Red DND-99 (L7528, Invitrogen) for 40 min, washed and then stained with 5 μM Calcein-AM (Thermofisher, C3100MP) diluted in fresh medium. Subsequently, cells treated with either DMSO, 1 μM Auranofin, 0.25 μM Salinomycin, 0.75 μM D9 or 0.0025 μM Cu. Imaging was performed using a Zeiss confocal microscope (Carl Zeiss, Baden-Württemberg, Germany) in intervals of 2 h.

### Synergy analysis

Dissociated organoids were plated with 5000 single cells per well in white 96-well plates with clear bottoms (Corning 2610). After incubation for 48 h to allow the formation of small organoids, cells were treated with increasing concentrations of the corresponding drugs, forming a matrix. Cell viability and death were measured as described above. Treatments were performed with at least three biological replicates and the average cell viability was used to calculate the score using the Bliss model with the SyngergyFinder platform^53^. In this analysis, scores <−5 were considered antagonistic, from −5 to 5 additive, and >5 synergistic.

### Glutathione detection

Dissociated LU-NB organoids were seeded in T25 flasks and incubated for 48 h before treatment and then treated for 48 h. Organoids were collected, washed with ice-cold PBS, resuspended in ice-cold 5% sulfosalicylic, and homogenized by sonication. Glutathione was measured using the Glutathione Colorimetric Detection kit (Invitrogen, EIAGSHC) according to protocol and detected with a Synergy2 plate reader (BioTek).

### siRNA transfection and western blot

Dissociated LU-NB-1 cells were seeded into a 6-well plate (5x105 cells/well) and incubated overnight. Cells were then transfected with either mock siRNA (Dharmacon) or different siRNAs targeting GPX4 (cat#: J-011676-05-0005; siRNA#1: 5′-ACGUCAAAUUCGAUAUGUU-3′, siRNA#2: 5′-GCUGCGUGGUGAAGCGCUA-3′) (Dharmacon). After overnight transfection, cells were resuspended and used for viability assay, as previously described, and western blotting. For western blot analysis, cells were collected after 72 h of transfection and lysed in RIPA buffer with protease inhibitor (Roche) and phosSTOP (Roche). Proteins were separated using SDS–polyacrylamide gel electrophoresis gels and transferred to nitrocellulose membranes TM(Bio-Rad, #1704271). Antibodies used were anti-GPX4 (Cell Signaling Technology, #59735) and anti-GAPDH (R&D Systems, #2275-PC-100). Imaging was performed using Luminata Forte Western HRP substrate (Millipore Sigma) and Amersham Imager 600 (GE Healthcare Bio-Sciences AB).

### Bulk RNA extraction

Snap-frozen tumor pieces were added to RNAlater-ICE (Invitrogen, #AM7030) and kept overnight at −20 °C before extraction. RNA was extracted from tumors and organoids using the AllPrep DNA/RNA Mini Kit (Qiagen, #80204).

### RNA-seq and analysis

Library preparation and mRNA sequencing were performed by the Center for Translational Genomic, Lund University. mRNA library preparation was performed using Illumina Stranded mRNA Prep, Ligation (Illumina, #20040534) and TruSeq Stranded mRNA Library Prep (Illumina, #20020594) kits on the King Fisher FLEX system (Thermo Fisher Scientific, #18-5400620). Sequencing was done using the NovaSeq 6000 SP Reagent Kit v1.5 (200 cycles; Illumina, #20040719) and the NovaSeq 6000 S1 Reagent Kit v1.5 (200 cycles; Illumina, #20028318) on the NovaSeq 6000 System (Illumina, #20012850). Reads were aligned to the human GRCh38 reference genome (Ensembl) using STAR^96^, and transcript summarization was carried out using featureCounts with subread package^97,98^ and the annotation (GTF) from Gencode version 33. Additionally, we conducted pseudoalignment using Kallisto^99^ with both non-coding RNA (ncRNA) and cDNA retrieved from the Ensembl repository release 109^100^. The summarization of Kallisto output was carried out using the tximport R package^101^, utilizing a transcript to gene table obtained from biomaRt^102^. Normalization was performed using the DESeq2 R package^103^.

RNA-seq analyses, both from public data and project-specific data, were performed either on the R2 Genomics Analysis and Visualization Platform (https://r2.amc.nl) or using R. The SEQC dataset^29^, Kocak dataset^31^, and the Versteeg dataset^30^, available at the R2 Genomics Platform, were used. Gene expression was determined as reads per millions (RPM) and transformed using log2(RPM) for single gene analysis and using z-score of log2(RPM) for signature analysis. Overall survival probability was analyzed through Kaplan Meier curves (median cut) and significance calculated through log rank analysis. Expression values were standardized for display in boxplots, and the significance of their difference between patients displaying different clinical features (i.e., *MYCN* amplification and resistance status) was computed using Welch’s test. The correlation between gene expression and age at diagnosis was determined using the “correlation with track” tool in R2. Spearman correlations between log2 transformed expression and age at diagnosis were computed for each gene in all the patients of the SEQC cohort. A total of 8554 genes presenting a significantly positive (*n* = 4572) or negative (*n* = 3982) correlation with aging were selected for further analysis. A Benjamini-Hochberg FDR threshold of 0.01 marked significance. Genes in the pathways of interest were computed from those pathways that exhibited enrichment for genes with high expression in significant association with better or worse survival and enrichment for genes with significant negative or positive correlation with age at diagnosis. Significance was estimated with one-tail Fisher’s exact tests corrected using the Benjamini-Hochberg approach.

The PDX dataset (Neuroblastoma COJEC in vivoPDX123) and PDX-derived organoids dataset (Neuroblastoma COJEC_PDX3 organoids) from previous work^12^ and from the current project for organoids (Neuroblastoma Auranofin Comb PDX1andPDX3R_org) and in vivo (NB_Aur-COJEC_PDX1_invivo) are publicly available in the R2 Genomics platform (https://hgserver1.amc.nl/). Analysis and visualization of heatmaps and differentially expressed genes (DEGs) were done using R2, FDR < 0.01. Heatmaps present the z-score transformation of the log2(RPM) for each gene in each sample, and gene hierarchical clustering was performed using Euclidean distance. Box plots for genetic signatures present the average z-score values over the gene set for each sample. Welch’s t test correction was used to analyze statistical differences between groups for individual signatures. Enrichment analyses were performed using Enrichr^104,105^ and Metascape^106^. Gene Set Enrichment Analysis (GSEA) was conducted, where differentially expressed genes were obtained for each comparison. Ranked files were created by ordering the DGE table using a ranking column, calculated as the product of -log10(*p* value) * sign(log_2_FC). The GSEA function from the ClusterProfiler R package^107^ was utilized, employing MSigDB Hallmark gene sets from the msigdbr R package^108^.

### Single cell data analysis

The expression of genes in the pathways of interest was evaluated in different NB cell populations. To this end, the processed single-nuclei data from Bedoya-Reina et al.^17^ were obtained. Using this dataset, a signature score for each pathway was calculated using Scanpy^109^. This score estimates the average expression of genes in the pathway of interest and subtracts the average expression of a random group of genes. In this way, a higher score suggests a higher average expression of genes in the pathway of interest than expected by change.

Single-nucleus RNA sequencing data of neoplastic cells from Yu et al.^32^ were retrieved from the CELLxGENE database. Data were downloaded as.h5ad files and imported into R using the anndata package. Expression matrices were transposed to a genes-by-cells format and metadata were extracted from the obs object. Seurat objects were created for the complete dataset and the neoplastic cells. Dimensional reduction coordinates (UMAP, PCA) were imported from the original dataset and incorporated into the respective Seurat objects. Data was normalized using the LogNormalize method with a scaling factor of 10,000. Visualizations were generated using Seurat and SeuratExtend functions^110,111^, specifically DimPlot2() for UMAP projections and DotPlot() for expression patterns of selected genes.

### Spatial transcriptomic data analysis

The expression of antioxidant pathways and related genes were analyzed using a spatial transcriptomic dataset from two high-risk NB patients^33^. Both patients were treated with the Rapid COJEC chemotherapy protocol, and tumor samples were obtained pre-therapy and post-therapy. Antioxidant pathways signatures (Supplementary data S1) were scored on the Visium gene expression data, using UCell^112^, rank-based signature enrichment analysis method.

### Statistical analyses

Statistical analyses pertinent to RNA were performed as described in the corresponding method sections. The remaining analyses were performed using GraphPad Prism 9. For tumor volume comparison, ordinary one-way ANOVA was performed with correction for multiple comparisons. Tumor growth curves were compared using two-way ANOVA followed by multiple comparison analysis with correction. The thresholds for the AUC (area under the curve) values were AUC < 5 for viability and AUC > 100 for cell death. Overall, a *p*-value < 0.05 was considered significant. Specific tests are included in the figure legends.

## Supplementary information

Supplementary Information

Manas et al_Supplementary data S1 - Gene Signatures

## Data Availability

Normalized RNA-seq data for the sets developed in this study has been publicly deposited at the R2 Genomic Analysis and Visualization Platform (http://r2.amc.nl) under the dataset names: NB_Aur-COJEC_PDX1_invivo (for in vivo data) and Neuroblastoma Auranofin Comb PDX1andPDX3R_org (for organoid data). All previously publicly available datasets and code have been properly referenced. All data needed to evaluate the conclusions in the paper is present in the paper and/or the Supplementary Materials.
