# Supplementary material for: Targeted ferroptosis induction enhances chemotherapy efficacy in chemoresistant neuroblastoma
Source: NPJ Precis Oncol. 2025 Sep 16;9:311. doi: 10.1038/s41698-025-01090-6 (PMC12441132; doi:10.1038/s41698-025-01090-6)

Table S1. Details of the LU-NB PDX and organoid models.

PDX Model	Origin	Stage	MYCN Status	Treatment Status	Patient Chemoresponse	PDX Chemoresponse	Organoid Model
PDX1	Primary patient tumor	IV undifferentiated	Amplified	Naive	Refractory	Refractory	LU-NB-1
PDX2	Metastatic patient tumor	IV undifferentiated	Amplified	Treated	Responsive	Responsive	LU-NB-2
PDX3	Primary patient tumor	III poorly differentiated	Amplified	Naive	Responsive	Responsive	LU-NB-3
PDX3-R	Relapsed PDX3-derived tumor	N/A undifferentiated	Amplified	Treated	N/A	Partially responsive	LU-NB-3R

Table S2. Evaluation of ferroptosis-inducing agents tested in organoids from each HR-NB PDX model. Data are presented as area under the curve (AUC) for viability (V) and cell death (CD).

	LU-NB-1		LU-NB-2		LU-NB-3		LU-NB-3R	
**Drug**	AUC (V)	AUC (CD)	AUC (V)	AUC (CD)	AUC (V)	AUC (CD)	AUC (V)	AUC (CD)
Altretamine	9,22	15,81	10,90	26,89	12,30	78,54	10,20	11,20
Artesunate	6,76	160,40	6,89	122,40	4,51	236,40	10,05	31,52
Auranofin	2,98	316,80	4,20	237,40	1,26	216,20	3,23	329,40
BSO	9,57	18,43	10,31	20,37	10,21	51,95	9,90	12,43
Cisplatin	8,39	95,42	8,18	102,60	2,54	464,60	7,26	88,76
DHA	9,88	31,87	9,31	36,29	9,88	85,15	10,43	12,33
Erastin	4,55	180,00	7,48	60,10	2,22	386,00	7,96	28,18
FIN56	9,66	14,34	10,55	26,79	9,00	88,31	8,86	15,67
Fluvastatin	8,20	17,01	10,39	29,48	9,08	80,01	9,64	17,57
Lovastatin	9,25	19,09	9,67	34,50	9,04	70,11	10,09	14,57
ML162	3,06	277,60	4,97	144,00	3,27	376,70	4,46	274,50
ML210	8,90	18,15	10,35	25,79	9,59	110,80	10,22	10,75
RSL3	3,66	308,50	5,92	121,30	2,83	247,20	0,79	569,60
Salinomycin	1,17	171,60	2,20	117,20	0,90	427,80	2,65	265,40
SAS	7,97	16,52	9,35	29,26	10,15	76,92	9,79	13,18
Sorafenib	6,34	128,10	10,48	22,68	10,97	40,37	10,97	13,45

Figure S1

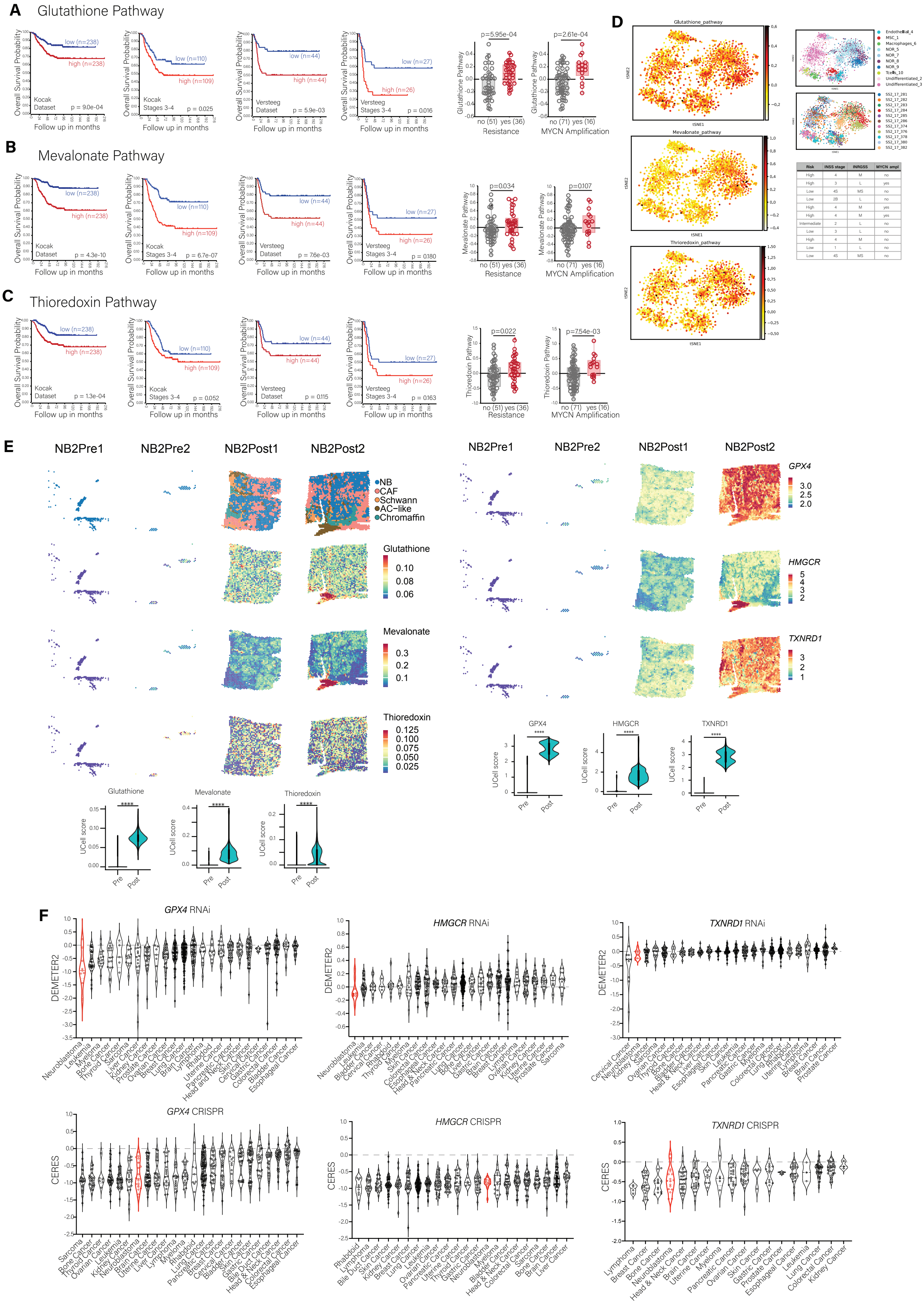
A Glutathione Pathway
B Mevalonate Pathway
C Thioredoxin Pathway
Overall Survival Probability
Follow up in months
low (n=238)
high (n=238)
Kocak Dataset
low (n=110)
high (n=109)
Kocak Stages 3-4
low (n=44)
high (n=44)
Versteeg Dataset
low (n=27)
high (n=26)
Versteeg Stages 3-4
p = 9.0e-04
p = 0.025
p = 5.9e-03
p = 0.016
p = 4.3e-10
p = 6.7e-07
p = 7.6e-03
p = 0.180
p = 1.3e-04
p = 0.052
p = 0.115
p = 0.163
Glutathione Pathway
Mevalonate Pathway
Thioredoxin Pathway
p=5.95e-04
p=2.61e-04
p=0.034
p=0.107
p=0.022
p=7.54e-03
no (51) yes (36)
Resistance
no (71) yes (16)
MYCN Amplification
D
Glutathione_pathway
Mevalonate_pathway
Thioredoxin_pathway
tSNE1
tSNE2
Risk
INSS stage
INRGSS
MYCN ampl
E
NB2Pre1
NB2Pre2
NB2Post1
NB2Post2
NB
CAF
Schwann
AC-like
Chromaffin
Glutathione
Mevalonate
Thioredoxin
GPX4
HMGCR
TXNRD1
UCell score
Pre
Post
F
GPX4 RNAi
HMGCR RNAi
TXNRD1 RNAi
DEMETER2
GPX4 CRISPR
HMGCR CRISPR
TXNRD1 CRISPR
CERES

Figure S1. RNA expression of ferroptosis-related antioxidant pathways. Analysis of the RNA expression of the Glutathione (**A**), Mevalonate (**B**) and Thioredoxin (**C**) pathways within the Kocak (n=476) and Versteeg (n=88) NB patient datasets. From left to right: Kaplan Meier overall survival curves for the full patient sets and for the high-risk patients (log rank statistical analysis); Box plots comparing RNA expression based on treatment resistance and MYCN amplification for the Versteeg dataset (t-test with Welch correction statistical analysis). **D)** Signature score for genes in the glutathione, mevalonate, and thioredoxin pathways, across the single nuclei analysis of eleven patients conducted by Bedoya-Reina et al. (2021). **E)** Spatial transcriptomic analysis of an NB patient tumor (NB2) pre- and post-chemotherapy (*33*). Samples were analyzed for the expression of genes involved in the glutathione pathway, mevalonate pathway, thioredoxin pathway, and *GPX4*, *HMGCR* and *TXNRD1*. NB = neuroblastoma tumor cells; CAF = cancer-associated fibroblasts; Schwann = Schwann cell-like; AC-like = adrenocortical-like Statistical analysis performed with unpaired Wilcoxon test, * p-value<0.05; **** p-value<0.0001. **F)** Gene dependency analysis performed using publicly available (DepMap Portal) cell line data from multiple cancers from RNA interference (RNAi) and CRISPR knockout assays. Gene analyzed: *GPX4, HMGCR*, and *TXNRD1*. Negative DEMETER2 and CERES scores indicate dependency.

Figure S2

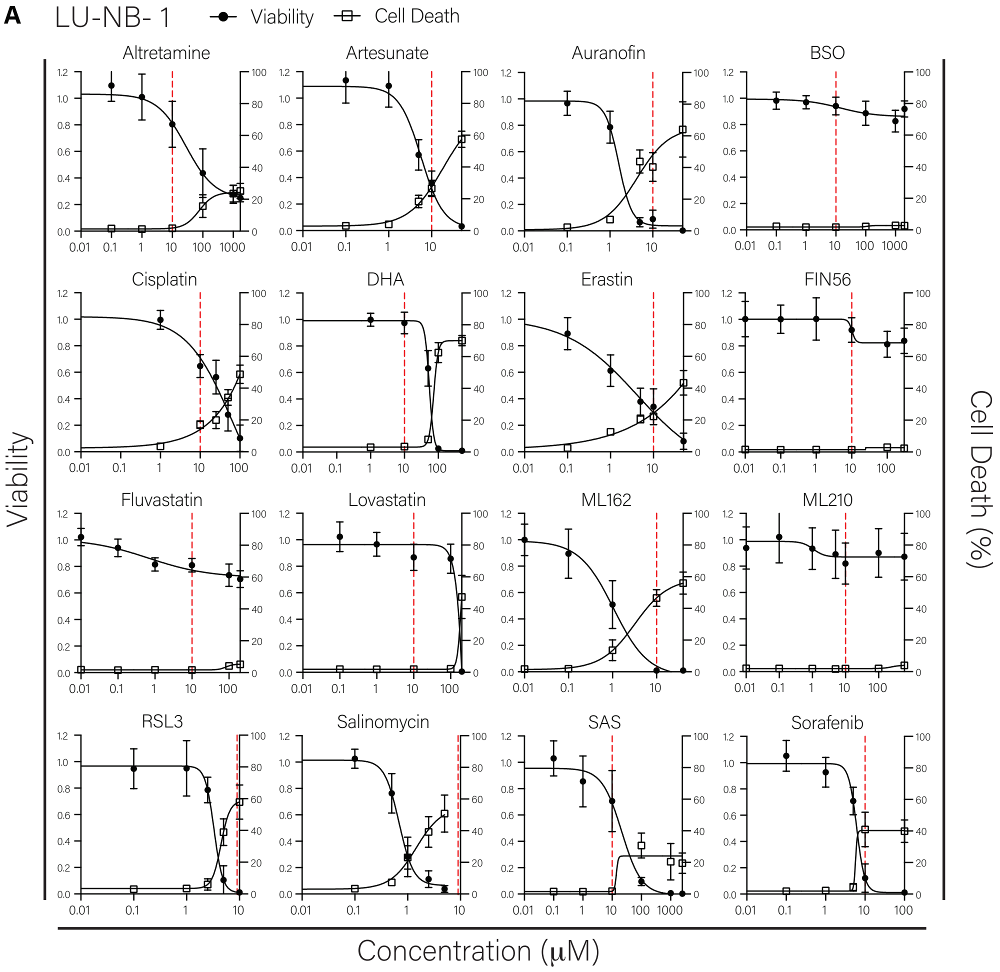
A
LU-NB- 1
Viability
Cell Death
Altretamine
Artesunate
Auranofin
BSO
Cisplatin
DHA
Erastin
FIN56
Fluvastatin
Lovastatin
ML162
ML210
RSL3
Salinomycin
SAS
Sorafenib
Viability
Cell Death (%)
Concentration (μM)

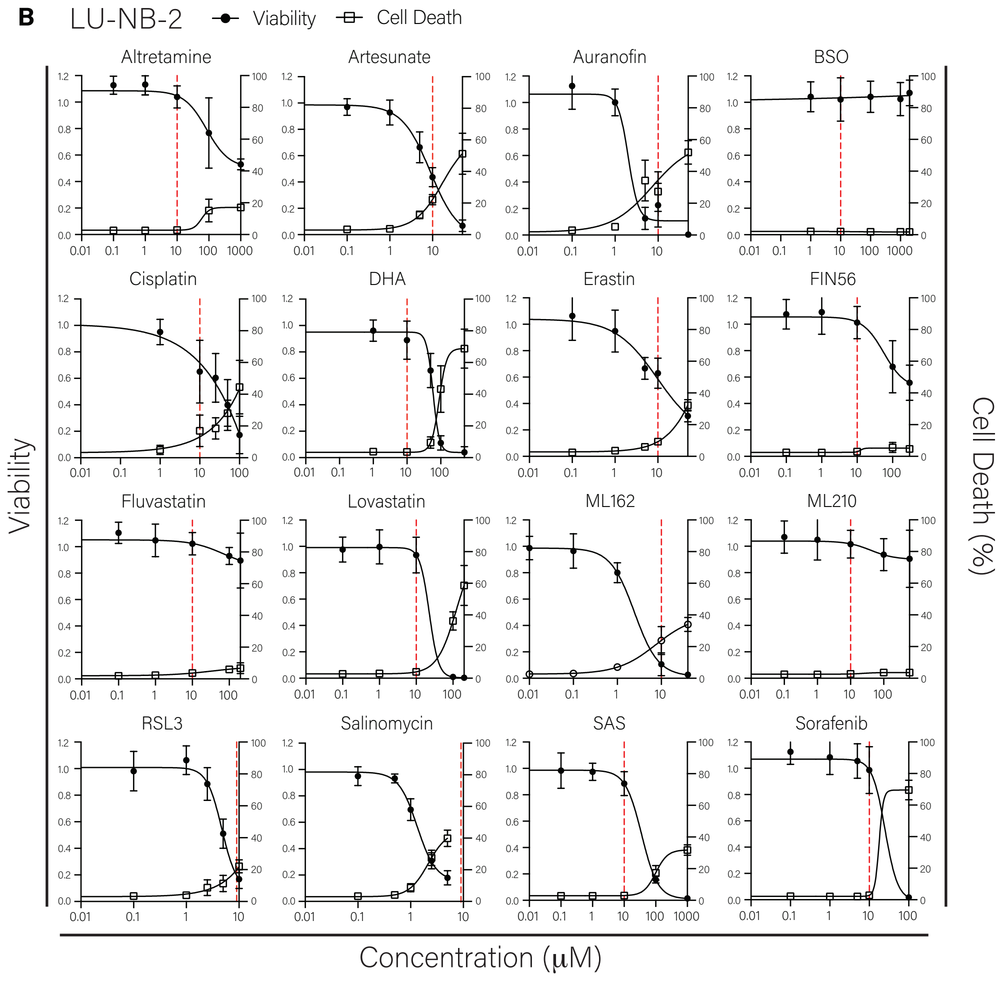
B
LU-NB-2
Viability
Cell Death
Altretamine
Artesunate
Auranofin
BSO
Cisplatin
DHA
Erastin
FIN56
Fluvastatin
Lovastatin
ML162
ML210
RSL3
Salinomycin
SAS
Sorafenib
Viability
Cell Death (%)
Concentration (μM)

C LU-NB-3 — Viability — Cell Death

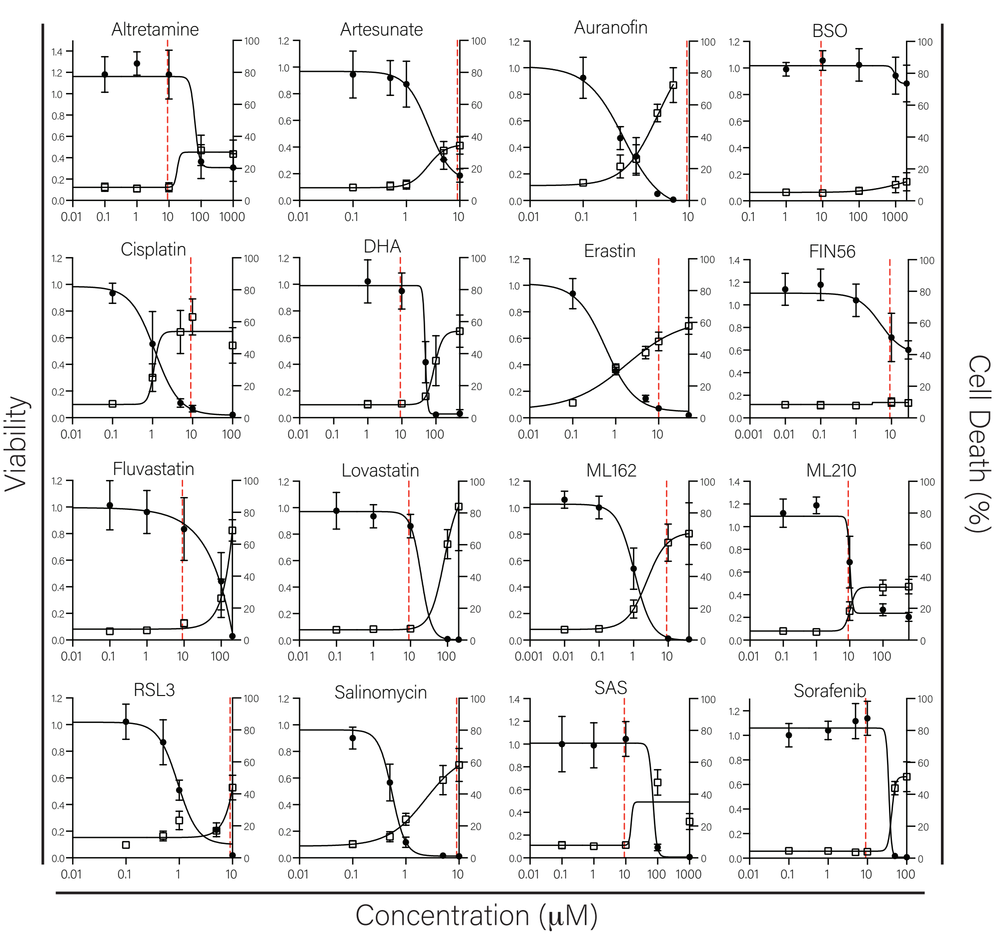

D LU-NB-3R — Viability — Cell Death

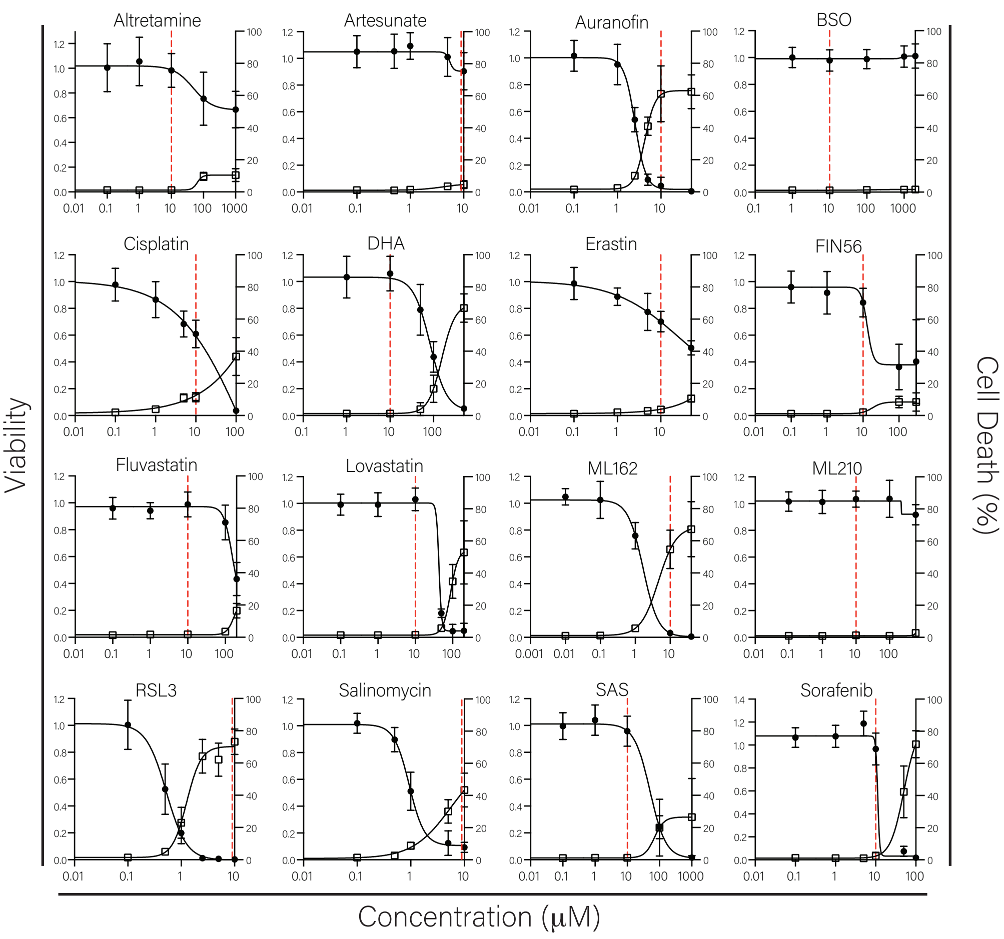

Figure S2. Screening of ferroptosis-inducing agents across NB patient-derived organoids. Individual drug plots of NB cell viability and cell death across PDX-derived organoids at 48h for the following compounds: Altretamine, Artesunate, Auranofin, Buthionine Sulfoximine (BSO), Cisplatin, DHA, Erastin, FIN56, Fluvastatin, Lovastatin, ML162, ML210, RSL3, Salinomycin, Sulfasalazine (SAS) and Sorafenib. **A**) LU-NB-1, **B)** LU-NB-2, **C)** LU-NB-3 and **D)** LU-NB-3R

Figure S3

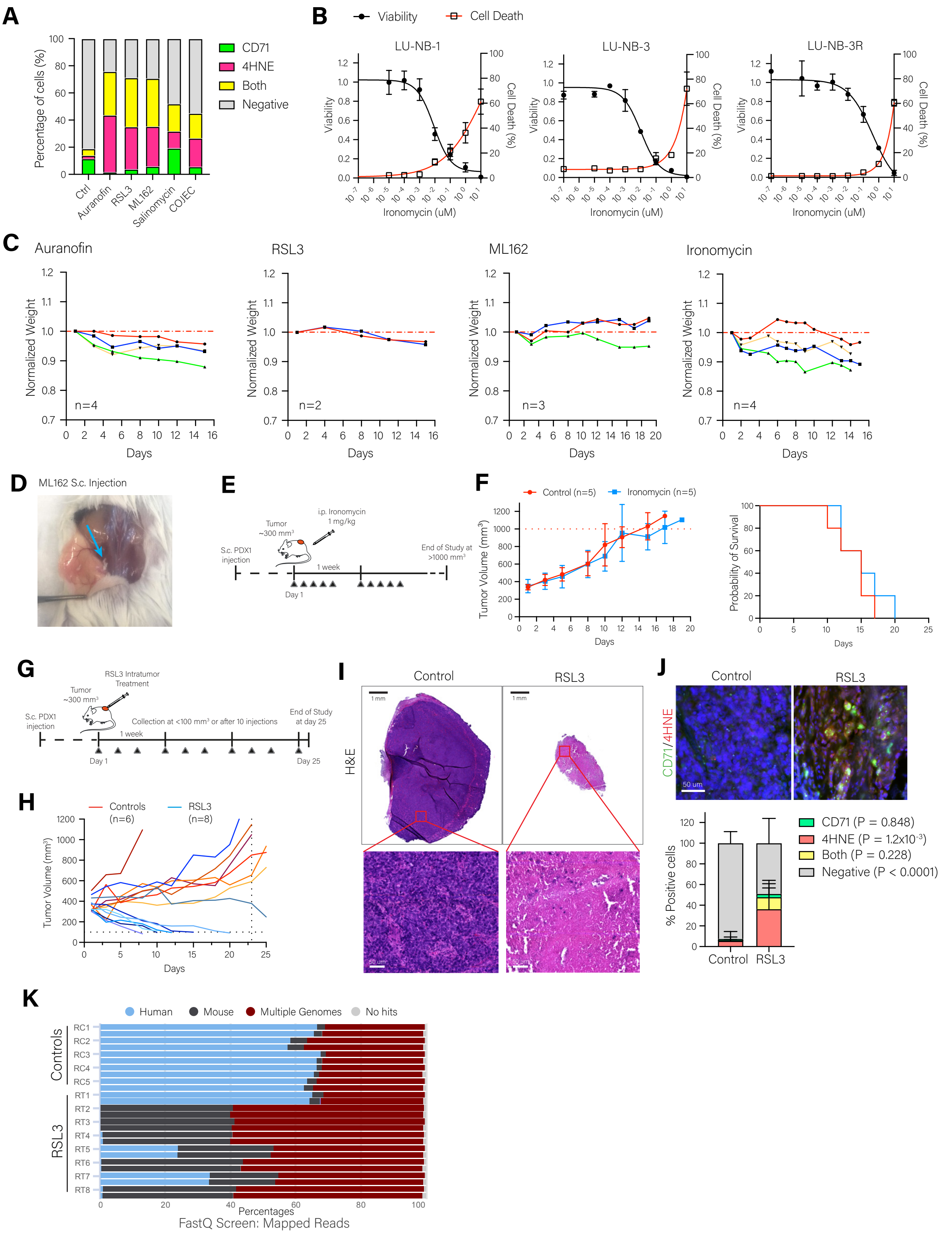
A
Percentage of cells (%)
CD71
4HNE
Both
Negative
Ctrl
Auranofin
RSL3
ML162
Salinomycin
COJEC
B
Viability
Cell Death
LU-NB-1
LU-NB-3
LU-NB-3R
Cell Death (%)
Ironomycin (uM)
C
Auranofin
RSL3
ML162
Ironomycin
Normalized Weight
n=4
n=2
n=3
n=4
Days
D
ML162 S.c. Injection
E
S.c. PDX1 injection
Tumor ~300 mm³
i.p. Ironomycin 1 mg/kg
1 week
Day 1
End of Study at >1000 mm³
F
Control (n=5)
Ironomycin (n=5)
Tumor Volume (mm³)
Days
Probability of Survival
Days
G
S.c. PDX1 injection
Tumor ~300 mm³
RSL3 Intratumor Treatment
Collection at <100 mm³ or after 10 injections
1 week
Day 1
End of Study at day 25
Day 25
H
Controls (n=6)
RSL3 (n=8)
Tumor Volume (mm³)
Days
I
Control
RSL3
H&E
1 mm
50 um
J
Control
RSL3
CD71/4HNE
50 um
% Positive cells
CD71 (P = 0.848)
4HNE (P = 1.2x10⁻³)
Both (P = 0.228)
Negative (P < 0.0001)
Control
RSL3
K
Human
Mouse
Multiple Genomes
No hits
Controls
RSL3
RC1
RC2
RC3
RC4
RC5
RT1
RT2
RT3
RT4
RT5
RT6
RT7
RT8
Percentages
FastQ Screen: Mapped Reads

Figure S3. Analysis of ferroptosis-inducing drugs in vitro and in vivo. A) Quantitation of the percentage of cells positive for CD71 and 4HNE for the experiment presented in Fig. 2C. **B)** NB cell viability (black) and cell death (red) curves for Ironomycin across three LU-NB PDX-derived organoid models. **C)** Normalized weight of NSG female mice treated with Auranofin, RSL3, ML162 and Ironomycin. **D)** Mouse injected subcutaneously (S.c.) with ML162 (1 mg/200 μl) 3 times/week for 19 days. Blue arrow indicates drug accumulation in the form of white powder. **E)** Schematic summary of the experimental design for Ironomycin (1 mg/kg) in vivo treatment. **F)** Normalized tumor growth curves and Kaplan Meier survival curves for experiment described in (E). Treatment groups: Control (n=5, red), Ironomycin (n=5, blue). Log rank statistical analysis for survival. **G)** Schematic summary of the experimental design for RSL3 intratumor in vivo treatment. **H)** Tumor volume curves for the experiment described in (G). Treatment groups: Control (n=6, red), RSL3 (n=8, blue). **I)** Hematoxylin and Eosin (H&E) staining of representative tumors from (H). Scale bars 1 mm for top panels and 50 μm for bottom panels. **J)** Immunofluorescence analysis of ferroptosis markers CD71 and 4HNE in representative tumors from (H). CD71 = green, 4HNE = red, nuclear DAPI staining = blue. Quantitative analysis of the CD71 and 4HNE staining. Two-way ANOVA statistical analysis followed by multiple comparison with two-stage step-up correction. **K)** FastQ Screen Mapped Reads for RNA analysis. Percentage of RNA reads for each tumor (Controls = RC; RSL3 treated = RT) mapped against human genome (blue), mouse genome (black), both (burgundy), or none (grey).

Figure S4

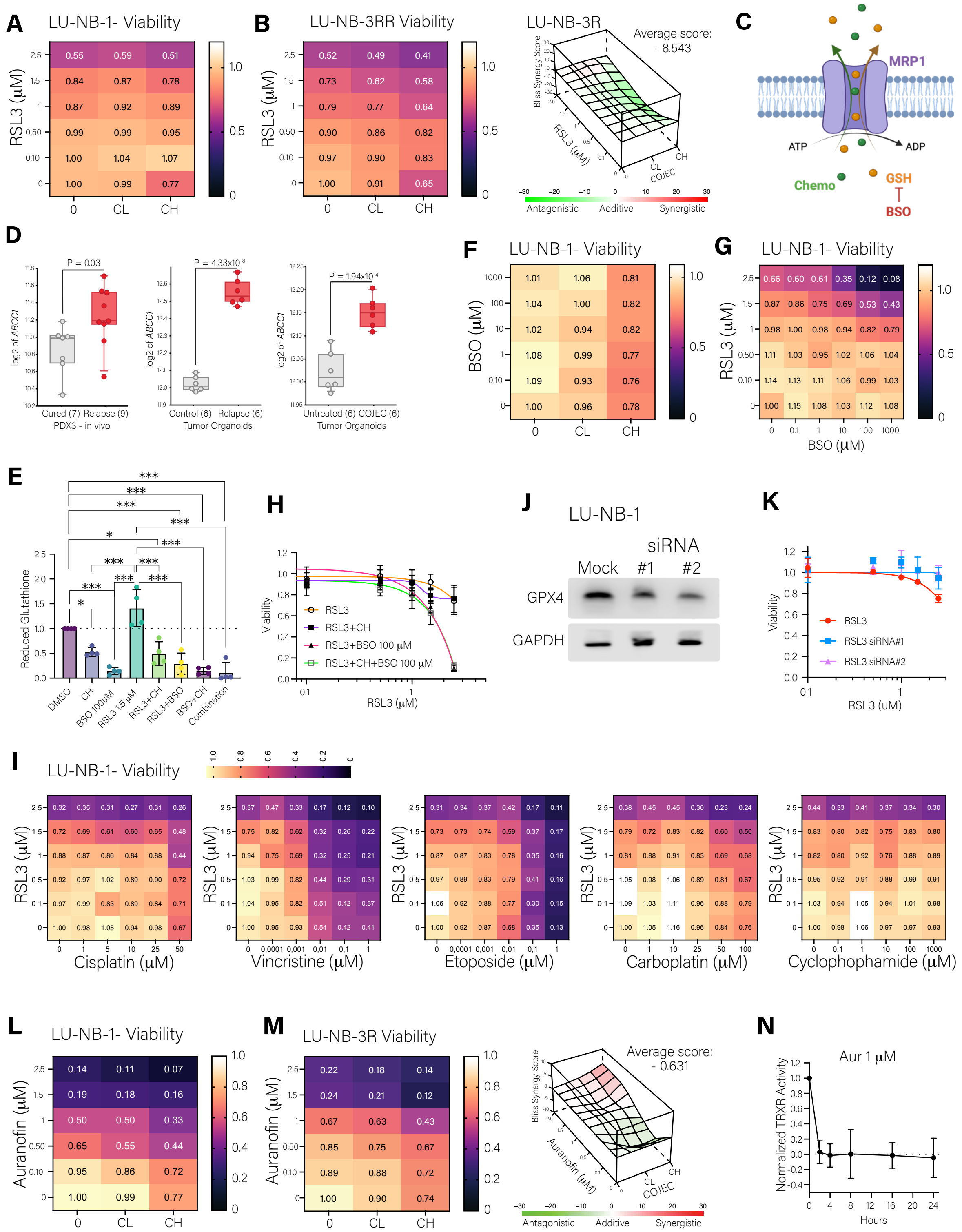
A
LU-NB-1- Viability
RSL3 (µM)
2.5
1.5
1
0.50
0.10
0
0.55 0.59 0.51
0.84 0.87 0.78
0.87 0.92 0.89
0.99 0.99 0.95
1.00 1.04 1.07
1.00 0.99 0.77
0
CL
CH
1.0
0.5
0
B
LU-NB-3RR Viability
RSL3 (µM)
0.52 0.49 0.41
0.73 0.62 0.58
0.79 0.77 0.64
0.90 0.86 0.82
0.97 0.90 0.83
1.00 0.91 0.65
LU-NB-3R
Average score:
- 8.543
Bliss Synergy Score
RSL3 (µM)
COJEC
CL
CH
Antagonistic
Additive
Synergistic
C
MRP1
ATP
ADP
Chemo
GSH
BSO
D
P = 0.03
log2 of ABCC1
Cured (7) Relapse (9)
PDX3 - in vivo
P = 4.33x10⁻⁸
Control (6) Relapse (6)
Tumor Organoids
P = 1.94x10⁻⁴
Untreated (6) COJEC (6)
Tumor Organoids
F
LU-NB-1- Viability
BSO (µM)
1000
100
10
1
0.10
0
1.01 1.06 0.81
1.04 1.00 0.82
1.02 0.94 0.82
1.08 0.99 0.77
1.09 0.93 0.76
1.00 0.96 0.78
0
CL
CH
G
LU-NB-1- Viability
RSL3 (µM)
0.66 0.60 0.61 0.35 0.12 0.08
0.87 0.86 0.75 0.69 0.53 0.43
0.98 1.00 0.98 0.94 0.82 0.79
1.11 1.03 0.95 1.02 1.06 1.04
1.14 1.13 1.11 1.06 0.99 1.03
1.00 1.15 1.08 1.03 1.12 1.08
0
0.1
1
10
100
1000
BSO (µM)
E
Reduced Glutathione
DMSO
CH
BSO 100uM
RSL3 1.5 µM
RSL3+CH
RSL3+BSO
BSO+CH
Combination
H
Viability
RSL3
RSL3+CH
RSL3+BSO 100 µM
RSL3+CH+BSO 100 µM
RSL3 (µM)
J
LU-NB-1
siRNA
Mock
#1
#2
GPX4
GAPDH
K
Viability
RSL3
RSL3 siRNA#1
RSL3 siRNA#2
RSL3 (uM)
I
LU-NB-1- Viability
RSL3 (µM)
Cisplatin (µM)
Vincristine (µM)
Etoposide (µM)
Carboplatin (µM)
Cyclophophamide (µM)
L
LU-NB-1- Viability
Auranofin (µM)
0.14 0.11 0.07
0.19 0.18 0.16
0.50 0.50 0.33
0.65 0.55 0.44
0.95 0.86 0.72
1.00 0.99 0.77
0
CL
CH
M
LU-NB-3R Viability
Auranofin (µM)
0.22 0.18 0.14
0.24 0.21 0.12
0.67 0.63 0.43
0.85 0.75 0.67
0.89 0.88 0.72
1.00 0.90 0.74
Average score:
- 0.631
Bliss Synergy Score
Auranofin (µM)
COJEC
Antagonistic
Additive
Synergistic
N
Aur 1 µM
Normalized TRXR Activity
Hours

Figure S4. Combination of ferroptosis-inducing agents with COJEC in NB organoids. A) Cell viability matrix of RSL3-COJEC combination treatment in LU-NB-1 organoids. CL= COJEC Low dose; CH= COJEC High dose. Scale represents loss of viability compared to control (black, 0) to high viability rate compared to control (white, >1.0). **B)** Cell viability matrix of RSL3-COJEC combination in LU-NB-3R organoids, and 3D representation of synergy analysis for the dose matrix. Bliss analysis performed with SynergyFinder. Negative scores (green) indicate antagonism, positive scores (red) indicate synergy, scores between -5 and 5 indicate additivity. Average overall score -8.543. **C)** Diagram of MRP1 mediated pumping of chemotherapy and GSH. BSO, buthionine sulfoximine**. D)** Comparison of ABCC1 (encoding MRP1) RNA expression across patient-derived models in vivo (PDX3 cured or relapsed) and in vitro (LU-NB-3 and LU-NB-3R organoids untreated or high COJEC-treated). T-test analysis with Welch correction. **E)** Evaluation of the intracellular level of reduced glutathione (GSH) in PDX1 organoids under different treatments (DMSO, CH = COJEC High, BSO 100 µM, RSL3 1.5 µM, the indicated combinations, or combination = RSL3 + CH + BSO) for 24 hours, normalized against the DMSO treated control. One-way ANOVA statistical analysis followed by multiple comparison with Tukey correction. (* P value<0.05, ** P value<0.01, *** P value<0.001). **F)** Cell viability matrix of BSO-COJEC combination in LU-NB-1 organoids. **G)** Cell viability matrix of RSL3-BSO combination in LU-NB-1 organoids. **H)** Viability curves of LU-NB-1 organoids treated with RSL3, COJEC, BSO, or combinations of these three. **I)** Cell viability matrixes of RSL3-Cisplatin, RSL3-Vincristine, RSL3-Etoposide, RSL3-Carboplatin and RSL3-Cyclophosphamide combinations in LU-NB-1 organoids. **J)** Western blot for LU-NB-1 organoids transfected with mock siRNA or GPX4 siRNAs #1 or #2 for 24h. GAPDH (glyceraldehyde-3-phosphate dehydrogenase) was used as loading control. **K)** Cell viability curves for LU-NB-1 organoids transfected with mock siRNA or GPX4 siRNAs #1 or #2 and treated with RSL3 for 48h. **L)** Cell viability matrix of Auranofin-COJEC combination in LU-NB-1 organoids. **M)** Cell viability matrix of Auranofin-COJEC combination in LU-NB-3R organoids, and 3D representation of synergy analysis for the dose matrix. Bliss analysis performed with SynergyFinder. Average overall score -0.631. **N)** Normalized TrxR enzymatic activity during time upon treatment with Auranofin 1 µM.

Figure S5

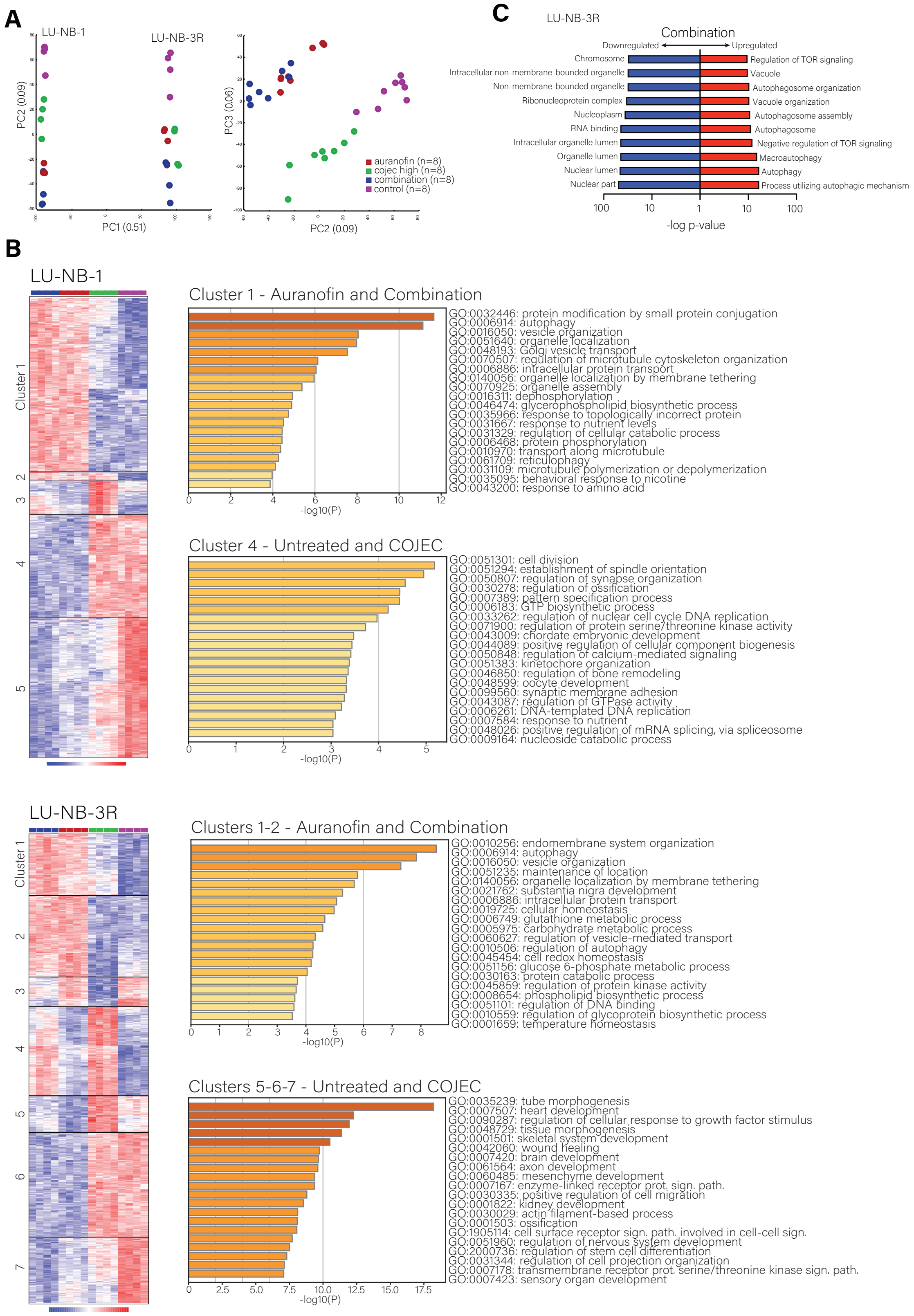
A
LU-NB-1
LU-NB-3R
PC2 (0.09)
PC1 (0.51)
PC3 (0.06)
PC2 (0.09)
auranofin (n=8)
cojec high (n=8)
combination (n=8)
control (n=8)
B
LU-NB-1
Cluster 1
2
3
4
5
Cluster 1 - Auranofin and Combination
GO:0032446: protein modification by small protein conjugation
GO:0006914: autophagy
GO:0016050: vesicle organization
GO:0051640: organelle localization
GO:0048193: Golgi vesicle transport
GO:0070507: regulation of microtubule cytoskeleton organization
GO:0006886: intracellular protein transport
GO:0140056: organelle localization by membrane tethering
GO:0070925: organelle assembly
GO:0016311: dephosphorylation
GO:0046474: glycerophospholipid biosynthetic process
GO:0035966: response to topologically incorrect protein
GO:0031667: response to nutrient levels
GO:0031329: regulation of cellular catabolic process
GO:0006468: protein phosphorylation
GO:0010970: transport along microtubule
GO:0061709: reticulophagy
GO:0031109: microtubule polymerization or depolymerization
GO:0035095: behavioral response to nicotine
GO:0043200: response to amino acid
0 2 4 6 8 10 12
-log10(P)
Cluster 4 - Untreated and COJEC
GO:0051301: cell division
GO:0051294: establishment of spindle orientation
GO:0050807: regulation of synapse organization
GO:0030278: regulation of ossification
GO:0007389: pattern specification process
GO:0006183: GTP biosynthetic process
GO:0033262: regulation of nuclear cell cycle DNA replication
GO:0071900: regulation of protein serine/threonine kinase activity
GO:0043009: chordate embryonic development
GO:0044089: positive regulation of cellular component biogenesis
GO:0050848: regulation of calcium-mediated signaling
GO:0051383: kinetochore organization
GO:0046850: regulation of bone remodeling
GO:0048599: oocyte development
GO:0099560: synaptic membrane adhesion
GO:0043087: regulation of GTPase activity
GO:0006261: DNA-templated DNA replication
GO:0007584: response to nutrient
GO:0048026: positive regulation of mRNA splicing, via spliceosome
GO:0009164: nucleoside catabolic process
0 1 2 3 4 5
-log10(P)
LU-NB-3R
Cluster 1
2
3
4
5
6
7
Clusters 1-2 - Auranofin and Combination
GO:0010256: endomembrane system organization
GO:0006914: autophagy
GO:0016050: vesicle organization
GO:0051235: maintenance of location
GO:0140056: organelle localization by membrane tethering
GO:0021762: substantia nigra development
GO:0006886: intracellular protein transport
GO:0019725: cellular homeostasis
GO:0006749: glutathione metabolic process
GO:0005975: carbohydrate metabolic process
GO:0060627: regulation of vesicle-mediated transport
GO:0010506: regulation of autophagy
GO:0045454: cell redox homeostasis
GO:0051156: glucose 6-phosphate metabolic process
GO:0030163: protein catabolic process
GO:0045859: regulation of protein kinase activity
GO:0008654: phospholipid biosynthetic process
GO:0051101: regulation of DNA binding
GO:0010559: regulation of glycoprotein biosynthetic process
GO:0001659: temperature homeostasis
0 1 2 3 4 5 6 7 8
-log10(P)
Clusters 5-6-7 - Untreated and COJEC
GO:0035239: tube morphogenesis
GO:0007507: heart development
GO:0090287: regulation of cellular response to growth factor stimulus
GO:0048729: tissue morphogenesis
GO:0001501: skeletal system development
GO:0042060: wound healing
GO:0007420: brain development
GO:0061564: axon development
GO:0060485: mesenchyme development
GO:0007167: enzyme-linked receptor prot. sign. path.
GO:0030335: positive regulation of cell migration
GO:0001822: kidney development
GO:0030029: actin filament-based process
GO:0001503: ossification
GO:1905114: cell surface receptor sign. path. involved in cell-cell sign.
GO:0051960: regulation of nervous system development
GO:2000736: regulation of stem cell differentiation
GO:0031344: regulation of cell projection organization
GO:0007178: transmembrane receptor prot. serine/threonine kinase sign. path.
GO:0007423: sensory organ development
0 2.5 5.0 7.5 10.0 12.5 15.0 17.5
-log10(P)
C
LU-NB-3R
Combination
Downregulated
Upregulated
Chromosome
Intracellular non-membrane-bounded organelle
Non-membrane-bounded organelle
Ribonucleoprotein complex
Nucleoplasm
RNA binding
Intracellular organelle lumen
Organelle lumen
Nuclear lumen
Nuclear part
Regulation of TOR signaling
Vacuole
Autophagosome organization
Vacuole organization
Autophagosome assembly
Autophagosome
Negative regulation of TOR signaling
Macroautophagy
Autophagy
Process utilizing autophagic mechanism
100 10 1 10 100
-log p-value

Figure S5. Transcriptome analysis (RNA-seq) of the Auranofin-COJEC combination treatment in vitro. A) Principal Component Analysis (PCA) plots. **B)** Heatmaps of gene expression (log2_z-score range -3 [blue] to 3 [red] and Euclidean distance clustering) and bar plots of Gene Ontology (GO) analysis for relevant clusters, for the unsupervised top 1000 most differentially expressed genes across treatments (Control, purple; COJEC, green; Auranofin, red; Combination, blue) for two organoid models: LU-NB-1 and LU-NB-3R. **C)** GO analysis of the supervised differentially expressed genes between Auranofin (1μM)-COJEC High combination treated LU-NB-3R organoids and controls. Red = Upregulated pathways in the combination group; Blue = Downregulated pathways in the combination group.

Figure S6

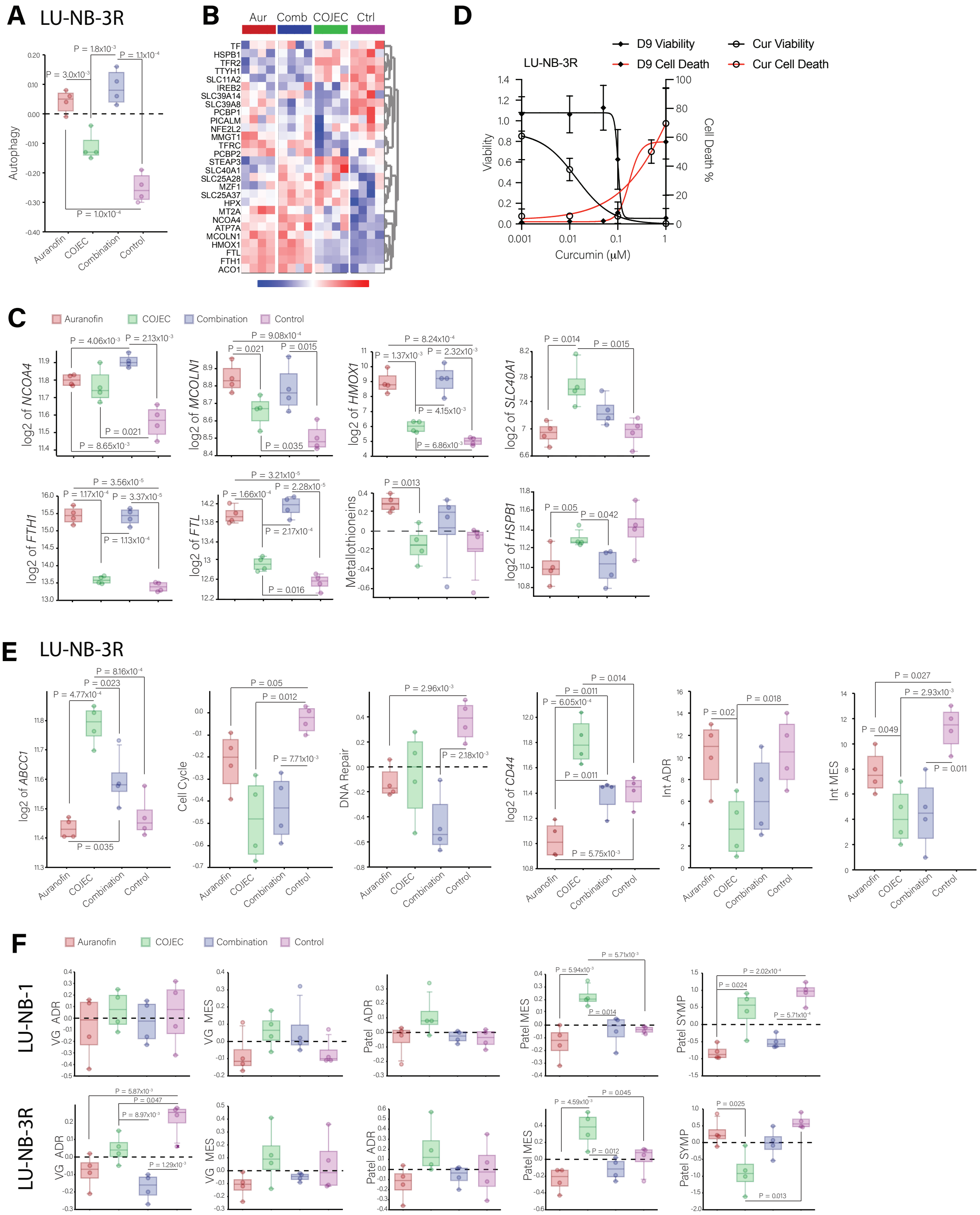
A
LU-NB-3R
Autophagy
Auranofin
COJEC
Combination
Control
B
Aur
Comb
COJEC
Ctrl
TF
HSPB1
TFR2
TTYH1
SLC11A2
IREB2
SLC39A14
SLC39A8
PCBP1
PICALM
NFE2L2
MMGT1
TFRC
PCBP2
STEAP3
SLC40A1
SLC25A28
MZF1
SLC25A37
HPX
MT2A
NCOA4
ATP7A
MCOLN1
HMOX1
FTL
FTH1
ACO1
D
LU-NB-3R
D9 Viability
D9 Cell Death
Cur Viability
Cur Cell Death
Viability
Cell Death %
Curcumin (μM)
C
Auranofin
COJEC
Combination
Control
Metallothioneins
E
LU-NB-3R
Cell Cycle
DNA Repair
Int ADR
Int MES
F
LU-NB-1
LU-NB-3R
VG ADR
VG MES
Patel ADR
Patel MES
Patel SYMP

Figure S6. Mechanism of action of Auranofin in NB organoids. A) RNA expression in LU-NB-3R of an autophagy gene signature across treatment conditions (Auranofin, COJEC, Combination, and Control). **B-C)** Supervised analysis of the RNA expression of iron metabolism genes across different treatment groups (log2_z-score range -3 [blue] to 3 [red] and Euclidean distance clustering in B; log2 expression in (C). One-way ANOVA statistical analysis followed by multiple comparison with Welch's correction. **D)** NB cell viability (black) and cell death (red) data of LU-NB-3R organoids treated with the TrxR inhibitors D9 and Curcumin (Cur). **E)** Supervised analysis of the RNA expression of relevant genes (log2 expression) and signatures (z-score) across treatment groups. One-way ANOVA statistical analysis followed by multiple comparison with Welch's correction. **F)** Supervised analysis of the RNA expression of the Patel et al. and Van Groningen et al. signatures across treatment groups (z-score) for LU-NB-1 and LU-NB-3R.

Figure S7

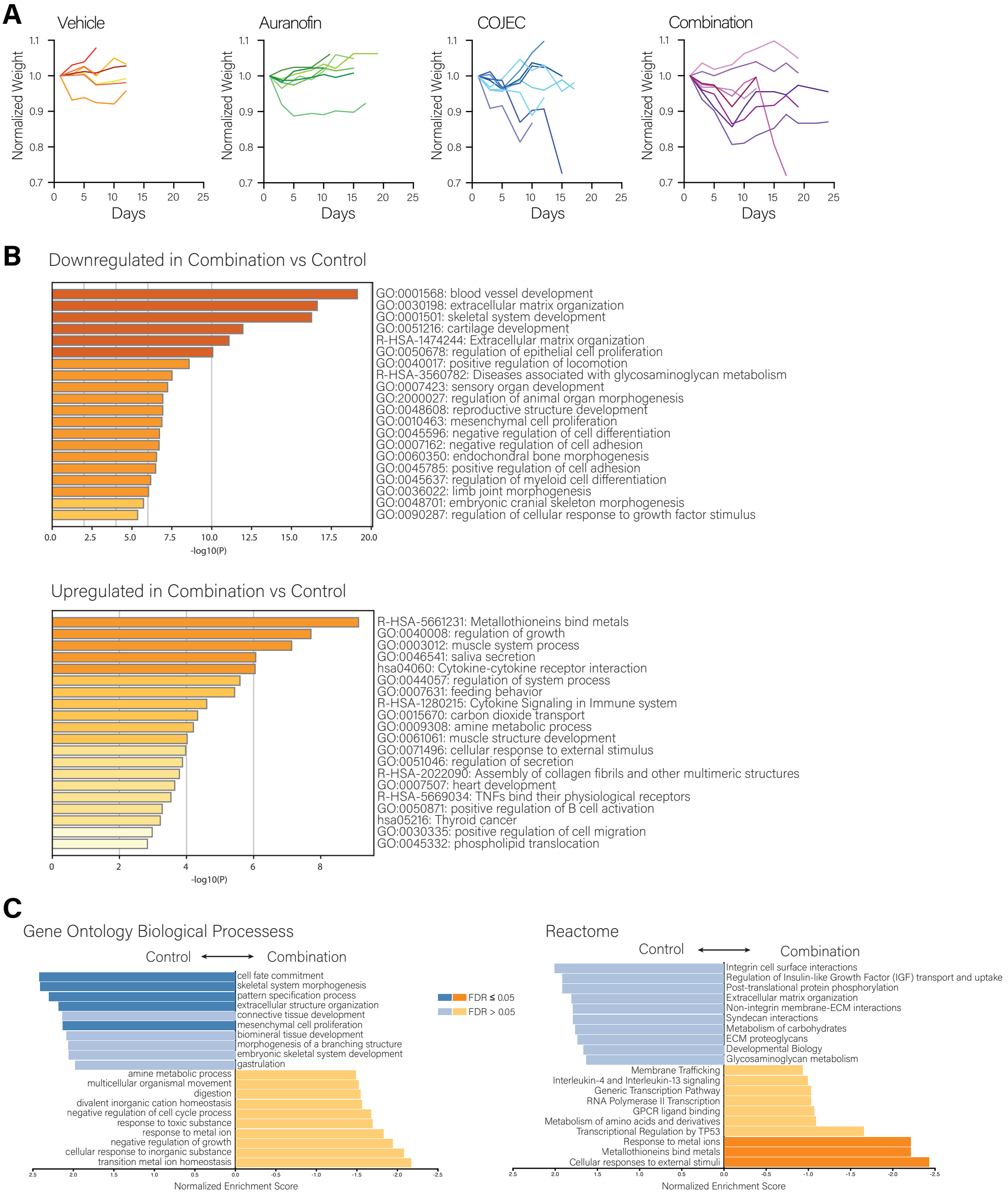

A
Vehicle
Auranofin
COJEC
Combination
Normalized Weight
Days
B
Downregulated in Combination vs Control
GO:0001568: blood vessel development
GO:0030198: extracellular matrix organization
GO:0001501: skeletal system development
GO:0051216: cartilage development
R-HSA-1474244: Extracellular matrix organization
GO:0050678: regulation of epithelial cell proliferation
GO:0040017: positive regulation of locomotion
R-HSA-3560782: Diseases associated with glycosaminoglycan metabolism
GO:0007423: sensory organ development
GO:2000027: regulation of animal organ morphogenesis
GO:0048608: reproductive structure development
GO:0010463: mesenchymal cell proliferation
GO:0045596: negative regulation of cell differentiation
GO:0007162: negative regulation of cell adhesion
GO:0060350: endochondral bone morphogenesis
GO:0045785: positive regulation of cell adhesion
GO:0045637: regulation of myeloid cell differentiation
GO:0036022: limb joint morphogenesis
GO:0048701: embryonic cranial skeleton morphogenesis
GO:0090287: regulation of cellular response to growth factor stimulus
-log10(P)
Upregulated in Combination vs Control
R-HSA-5661231: Metallothioneins bind metals
GO:0040008: regulation of growth
GO:0003012: muscle system process
GO:0046541: saliva secretion
hsa04060: Cytokine-cytokine receptor interaction
GO:0044057: regulation of system process
GO:0007631: feeding behavior
R-HSA-1280215: Cytokine Signaling in Immune system
GO:0015670: carbon dioxide transport
GO:0009308: amine metabolic process
GO:0061061: muscle structure development
GO:0071496: cellular response to external stimulus
GO:0051046: regulation of secretion
R-HSA-2022090: Assembly of collagen fibrils and other multimeric structures
GO:0007507: heart development
R-HSA-5669034: TNFs bind their physiological receptors
GO:0050871: positive regulation of B cell activation
hsa05216: Thyroid cancer
GO:0030335: positive regulation of cell migration
GO:0045332: phospholipid translocation
-log10(P)
C
Gene Ontology Biological Processess
Control
Combination
cell fate commitment
skeletal system morphogenesis
pattern specification process
extracellular structure organization
connective tissue development
mesenchymal cell proliferation
biomineral tissue development
morphogenesis of a branching structure
embryonic skeletal system development
gastrulation
amine metabolic process
multicellular organismal movement
digestion
divalent inorganic cation homeostasis
negative regulation of cell cycle process
response to toxic substance
response to metal ion
negative regulation of growth
cellular response to inorganic substance
transition metal ion homeostasis
FDR ≤ 0.05
FDR > 0.05
Normalized Enrichment Score
Reactome
Control
Combination
Integrin cell surface interactions
Regulation of Insulin-like Growth Factor (IGF) transport and uptake
Post-translational protein phosphorylation
Extracellular matrix organization
Non-integrin membrane-ECM interactions
Syndecan interactions
Metabolism of carbohydrates
ECM proteoglycans
Developmental Biology
Glycosaminoglycan metabolism
Membrane Trafficking
Interleukin-4 and Interleukin-13 signaling
Generic Transcription Pathway
RNA Polymerase II Transcription
GPCR ligand binding
Metabolism of amino acids and derivatives
Transcriptional Regulation by TP53
Response to metal ions
Metallothioneins bind metals
Cellular responses to external stimuli
Normalized Enrichment Score

Figure S7. Combination treatment with Auranofin-COJEC in the NB PDX1 model. A) Normalized weight of PDX1-bearing NSG female mice treated with Vehicle saline with 5% DMSO (orange), Auranofin (green), COJEC (blue), and Combination (purple). **B)** Top 20 most significant gene ontology terms enriched in either the Combination or Control groups when compared with each other (Metascape enrichment analysis). **C)** GSEA analysis of the combination group against the control group for GO “biological process” and Reactome, performed with the Webgestalt platform. Blue = gene sets enriched in the control group, Orange = gene sets enriched in the combination group.

Supplementary Data Files

Supplementary Data S1. Gene Signatures. Gene lists of the signatures used for mRNA analysis. Glutathione, Mevalonate, Thioredoxin, Autophagy, Iron metabolism, Metallothioneins, and ADR/MES signatures from Mañas et al. (Int), Patel el at. and Van Groningen et al. (See excel file.)

Supplementary Data S2. Raw Western Blot. Western blot membrane images corresponding to the western blot data presented in Figure S4J.

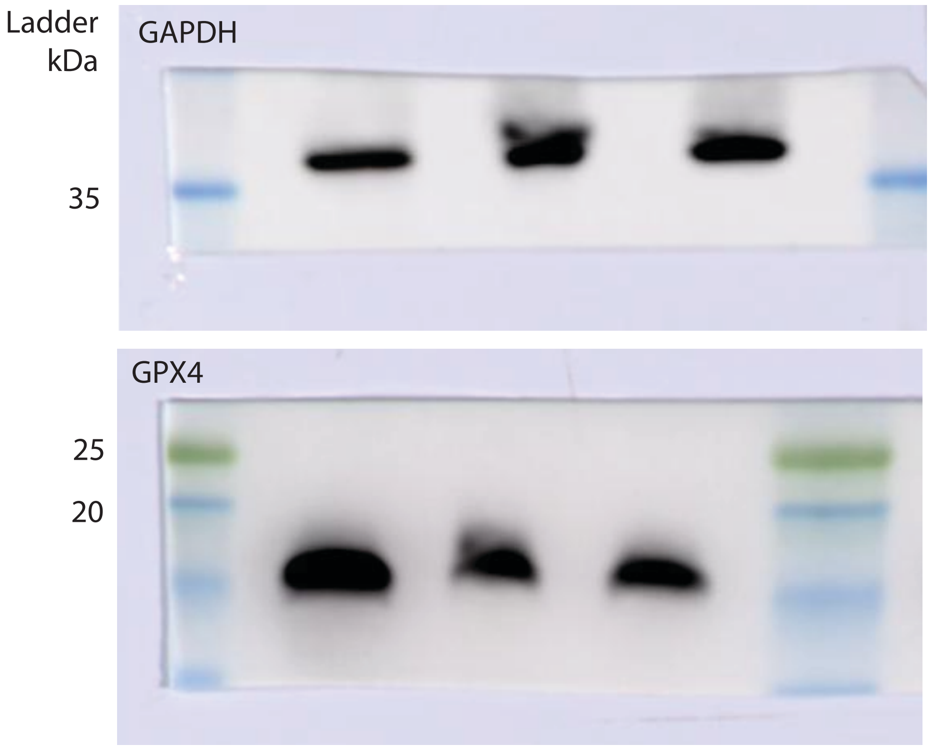

Supplement: Supplementary file 1 — Supplementary Information [file 41698_2025_1090_MOESM1_ESM.pdf]
